# Rapamycin administration is not a valid therapeutic strategy for every case of mitochondrial disease

**DOI:** 10.1016/j.ebiom.2019.03.025

**Published:** 2019-03-18

**Authors:** Eliana Barriocanal-Casado, Agustín Hidalgo-Gutiérrez, Nuno Raimundo, Pilar González-García, Darío Acuña-Castroviejo, Germaine Escames, Luis C. López

**Affiliations:** a Departamento de Fisiología, Facultad de Medicina, Universidad de Granada, 18016 Granada, Spain; b Instituto de Biotecnología, Centro de Investigación Biomédica, Universidad de Granada, 18016 Granada, Spain; c Centro de Investigación Biomédica en Red de Fragilidad y Envejecimiento Saludable (CIBERFES), Spain; d Universitätsmedizin Göttingen, Institute fur Zellbiochemie, Humboldtallee 23, room 01.423, 37073 Göttingen, Germany

**Keywords:** CoQ deficiency, Mitochondrial encephalopathy, Mitochondrial diseases, mTORC1, Mouse model

## Abstract

**Background:**

The vast majority of mitochondrial disorders have limited the clinical management to palliative care. Rapamycin has emerged as a potential therapeutic drug for mitochondrial diseases since it has shown therapeutic benefits in a few mouse models of mitochondrial disorders. However, the underlying therapeutic mechanism is unclear, the minimal effective dose needs to be defined and whether this therapy can be generally used is unknown.

**Methods:**

We have evaluated whether low and high doses of rapamycin administration may result in therapeutic effects in a mouse model (*Coq9*^*R239X*^) of mitochondrial encephalopathy due to CoQ deficiency. The evaluation involved phenotypic, molecular, image (histopathology and MRI), metabolomics, transcriptomics and bioenergetics analyses.

**Findings:**

Low dose of rapamycin induces metabolic changes in liver and transcriptomics modifications in midbrain. The high dose of rapamycin induces further changes in the transcriptomics profile in midbrain due to the general inhibition of mTORC1. However, neither low nor high dose of rapamycin were able to improve the mitochondrial bioenergetics, the brain injuries and the phenotypic characteristics of *Coq9*^*R239X*^ mice, resulting in the lack of efficacy for increasing the survival.

**Interpretation:**

These results may be due to the lack of microgliosis-derived neuroinflammation, the limitation to induce autophagy, or the need of a functional CoQ-junction. Therefore, the translation of rapamycin therapy into the clinic for patients with mitochondrial disorders requires, at least, the consideration of the particularities of each mitochondrial disease.

**Fund:**

Supported by the grants from “Fundación Isabel Gemio - Federación Española de Enfermedades Neuromusculares – Federación FEDER” (TSR-1), the NIH (P01HD080642) and the ERC (Stg-337327).

## Graphical abstract

**Figure:**
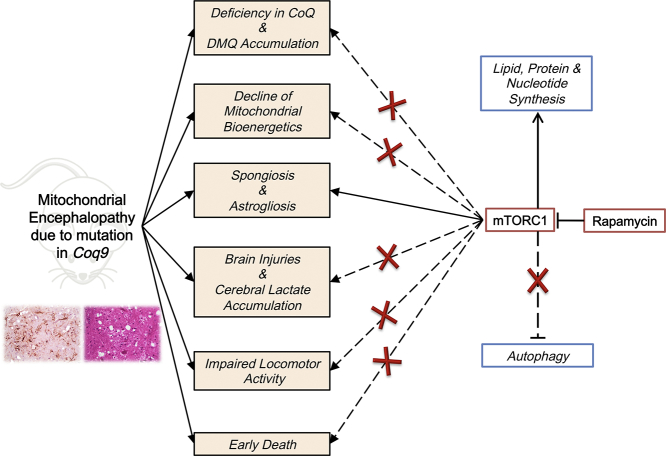

**Research in context**

**Evidence before this study**

Rapamycin therapy has shown therapeutic benefits in a few mouse models of mitochondrial diseases, opening the possibility of the translation of this therapeutic option into the clinic. However, a common therapeutic mechanism has not been identified and it is unclear whether rapamycin would be effective in a human equivalent dose or whether mTORC1 inhibition would be useful only in specific cases of mitochondrial dysfunction or in all cases of mitochondrial diseases.

**Added value of this study**

This study reports that rapamycin administration, at either low or high doses, does not result in therapeutic benefits in a mouse model of mitochondrial encephalopathy due to CoQ deficiency. The molecular, transcriptomics and metabolic analyses provide important insights about the dose-dependent and tissue-specific effects of rapamycin in vivo.

**Implications of all the available evidence**

These results are important for the translational perspective of rapamycin therapy, since it points out the limitation of this therapeutic strategy for some particular cases of mitochondrial diseases. The identification of specific mechanistic changes may contribute to the future definition of rapamycin therapy in patients with mitochondrial diseases.

Alt-text: Unlabelled Box

## 1 Introduction

Mitochondrial disorders comprise the most common group of inborn errors of metabolism and the therapeutic options for most of these diseases are limited to palliative care. However, a promising drug-based therapy was reported in 2013 in a mouse model of mitochondrial disease due to mitochondrial complex I deficiency [1]. In that study, Johnson and colleagues demonstrated that chronic administration of a high dose of rapamycin, a compound that inhibits a protein kinase called mechanistic target of rapamycin complex 1 (mTORC1), delays the onset and progression of neurological symptoms in a mouse model of Leigh syndrome and mitochondrial complex I deficiency due to the lack of the *Ndufs4* subunit of the mitochondrial complex I [1]. mTORC1 lies at the hub of cellular signaling sensing nutrient availability to regulate protein and lipid synthesis, translation, autophagy, and metabolism. However, the mechanism by which rapamycin delayed the progression of the disease in the *Ndufs4*^*−/−*^ mouse model was not clear since the evaluated pathways did not provide convincing results and the mutant mice still presented a severe mitochondrial dysfunction [1]. Moreover, the study of Johnson and colleagues [1] left opened two important questions about the therapeutic feasibility of rapamycin therapy in mitochondrial diseases: (1) whether rapamycin would be effective in a human equivalent dose (the equivalent dose used by Johnson and colleagues is much higher than the one used in human clinical trials to avoid side effects), which has been used, among others, in animal studies about aging [2]; and (2) whether mTORC1 inhibition would be useful only in cases of *Ndufs4* deficiency, in cases of complex I deficiency, in all cases of Leigh syndrome or mitochondrial encephalopathies or in all cases of mitochondrial diseases.

Other mitochondrial disorder different from Complex I deficiency is Coenzyme Q10 (CoQ_10_) deficiency syndrome (OMIM 607426), which is clinically manifested by five major phenotypes: encephalomyopathy, severe infantile multisystemic disease, nephropathy, cerebellar ataxia, and isolated myopathy [3]. The encephalomyopatic phenotype associated to CoQ deficiency has been mimicked in the *Coq9*^*R239X*^ mouse model, which has a reduction in the components of the Complex Q with the subsequent decrease in the levels of CoQ and accumulation of demethoxyubiquinone (DMQ); disruption in sulfide metabolism; increase in free complex III in the brain, leading to a decrease in mitochondrial respiration and ATP synthesis, as well as to an increase of oxidative stress; and severe reactive astrogliosis and spongiform degeneration with early death. Therefore, *Coq9*^*R239X*^ mice show clinical, histopathological, biochemical and molecular signs of encephalopathy, representing an excellent model to test therapies for mitochondrial diseases [4].

In this study, we evaluated whether low and high doses of rapamycin administration may result in therapeutic effects in a mouse model of mitochondrial encephalomyopathy due to CoQ deficiency. For that purpose, we studied the main pathways related to mTORC1 and mitochondrial metabolism and performed a more general transcriptomics and metabolomics profile. Moreover, we made a pilot study to test two drugs, trehalose [5] and PF-4708671 [6], that modulate autophagy or lipid synthesis and protein translation, respectively, two of the downstream pathways of mTORC1.

## 2 Material and methods

### 2.1 Mouse model and treatments

The *Coq9*^R239X^ mouse model of mitochondrial encephalomyopathy used in this study was generated and characterized under mix of C57BL/6N and C57BL/6J genetic backgrounds as reported previously [4,7]. *Coq9*^*R239X/+*^ mice were crossbred in order to generate *Coq9*^*+/+*^, *Coq9*^*R239X/+*^, *Coq9*^*R239X/R239X*^ (referred in the article as *Coq9*^R239X^). Only homozygous wild-type and mutant mice were used in the study. All experiments were performed according to a protocol approved by the Institutional Animal Care and Use Committee of the University of Granada (procedure CEEA 2014-64) and were in accordance with the European Convention for the Protection of Vertebrate Animals used for Experimental and Other Scientific Purposes (CETS # 123) and the Spanish law (R.D. 53/2013). Mice were housed in the Animal Facility of the University of Granada under a specific pathogen free zone with lights on at 7:00 AM and off at 7:00 PM with unlimited access to water and rodent chow.

Rapamycin (from LC Labs, Woburn, MA) was microencapsulated by Southwest Research Institute (San Antonio, TX), using a spinning disk atomization coating process with the enteric coating material Eudragit S100 (Röhm Pharma, Germany) [2]. Standard mouse chow was ground to a powder and mixed with microencapsulated rapamycin at 28 or 225 ppm. 300 ml of 1% agar melted in sterile water was added per kilogram of powdered chow and the mixture was pelleted and baked at 55 °C for 2–3 h to harden. Pellets were stored at 4 °C (short-term storage) or −20 °C (long-term storage). Control food contained no drug but microencapsulation material (Eudragit S100) alone at a concentration matching that in the rapamycin chow, as indicated.

Trehalose was given to the mice in their drinking water at a concentration of 2% (*w*/*v*) [5]. PF-4708671 treatment was administered by daily intraperitoneal injection at a concentration of 35 mg/kg/d [8]. The drug was dissolved at 5 mg/ml in 40% PEG400, 27.5% PG, 0.25% Tween80 in PBS. This vehicle without PF-4708671 was also injected in *Coq9*^R239X^ mice as a control.

Mice began receiving the assigned treatments at 1 month of age and were euthanized at 3 months of age for the experimental assays. Animals were randomly assigned in experimental groups. Data were randomly collected and processed as well.

The body weight was collected throughout the treatment, before starting (0 months) and at 1 and 2 months after the treatment (what corresponds with 1, 2 and 3 months of age). The motor coordination was assessed using the rotarod test by recording length of time that mice could remain on the rod (“latency to fall”) up to 300 s [9].

### 2.2 Sample preparation and western blot analysis in tissues

Western blot analyses were performed in cerebrum, kidney, heart and liver as previously described [7]. Band quantification was carried out using an Image Station 2000R (Kodak, Spain) and a Kodak 1D 3.6 software. Protein band intensity was normalized to GAPDH, and the data expressed in terms of percent relative to wild-type mice.

The following primary antibodies were used: anti-phospho-S6 Ribosomal Protein (Ser235/236) (Cell Signaling, 4856S), anti-S6 Ribosomal Protein (Cell Signaling, 2217S), anti-SQSTM1/p62 (Proteintech, 18420-1-AP), anti-LC3 (Novusbio, NB100–2220), and anti-GAPDH (Santa Cruz Biotechnology, sc-166574).

### 2.3 Quantification of CoQ_9_ and CoQ_10_ levels in mice tissues

After lipid extraction from homogenized tissues, CoQ_9_ and CoQ_10_ levels were determined via reversed-phase HPLC coupled to electrochemical (EC) detection [4,7]. The results were expressed in ng CoQ/mg prot.

### 2.4 Subcellular fractionation and CoQ-dependent respiratory chain activities

Mitochondrial isolation was performed as previously described [10]. After tissues homogenization, brain and kidney homogenates were centrifuged at 1000 ×*g* for 5 min at 4 °C to remove nuclei and debris. Mitochondria were collected from supernatants after centrifuging at 14,400 ×*g* for 2 min at 4 °C (twice). The final crude mitochondrial pellet was store at −80 °C [11].

CoQ dependent respiratory chain activities were measured in submitochondrial particles. To prepare submitochondrial particles, each mitochondrial pellet (100 μg prots) was suspended and sonicated in 100 μl of 0.1 M potassium phosphate buffer, pH 7.5. Complex I + III activity was measured at 30 °C in the presence of 0.5 mM potassium cyanide, 0.2 mM NADH and 0.1 mM cytochrome *c*, as the rotenone-sensitive reduction of cytochrome *c* at 550 nm [11]. The results were expressed in percentage relative to the wild type. Complex II + III activity was measured at 30 °C in the presence of 0.5 mM KCN, 0.3 mM succinate and 0.01 mM rotenone. The reaction was initiated by addition of 0.1 mM cytochrome *c* and decrease in absorbance was monitored at 550 nm. The results were expressed in percentage relative to the wild type.

### 2.5 Mitochondrial respiration

To isolate fresh mitochondria, mice were sacrificed and the organs were immediately extracted and placed in ice. The final crude mitochondrial pellet was re-suspended in MAS 1× medium [7,9]. Mitochondrial respiration was measured by using an XFe24 Extracellular Flux Analyzer (Seahorse Bioscience) [12]. Respiration by the mitochondria was sequentially measured in a coupled state with the substrates (succinate, malate, glutamate, and pyruvate) present (basal respiration or State 2), followed by State 3o (phosphorylating respiration, in the presence of ADP and substrates); State 4 (non-phosphorylating or resting respiration) was measured after addition of oligomycin when all ADP was consumed, and then maximal uncoupler-stimulated respiration (State 3u) [7,9]. All data were expressed in pmol/min/mg protein.

### 2.6 Histology and immunohistochemistry

Mice brains were formalin fixed and paraffin embedded. Multiple sections (4 μm thickness) were deparaffinized with xylene and stained with hematoxylin and eosin (H&E). Immunohistochemistry was carried out in the same sections, using the following primary antibodies: Glial fibrillary acidic protein or anti-GFAP (Millipore, MAB360), and anti-Iba-1 (Wako, 019-19741). Dako Animal Research Kit for mouse primary antibodies (Dako Diagnóstico S.A., Spain) was used for the qualitative identification of antigens by light microscopy. Sections were examined at 40–400 magnifications with an OLYMPUS CX41 microscope, and the images were scanned under equal light conditions with the CELL A computer program [4]. The GFAP positive signal was quantified by using the software ImageJ (National Institutes of Health, USA) and the results were expressed by the percentage of the positive signal.

### 2.7 In vivo MRI and proton MRS

MRI and MRS studies were conducted using a 7T horizontal bore magnet Bruker Biospec TM 70/20 USR designed for small animal experimentation, as previously reported [9,13].

### 2.8 Transcriptome analysis by RNA-seq

For the RNA-Seq we dissected the midbrain of the animals. RNA was extracted following the TRI Reagent Solution protocol from Applied Biosystems. Library preparation and sequencing was performed at the Transcriptome and Genome Analysis Laboratory, NGS Core Unit at the Universitzy Medical Center Göttingen. Quality and integrity of RNA was assessed with the Fragment Analyzer from Advanced Analytical by using the standard sensitivity RNA Analysis Kit (DNF-471). All samples selected for sequencing exhibited an RNA integrity number over 8. RNA-seq libraries were performed using 500 ng total RNA of a non stranded RNA Seq, massively-parallel mRNA sequencing approach from Illumina (TruSeq RNA Library Preparation Kit v2, Set A; 48 samples, 12 indexes, Cat. NRS-122-2001).

For accurate quantitation of cDNA libraries a fluorometric based system, the QuantiFluor™dsDNA System from Promega were used. The size of final cDNA libraries was determined by using the dsDNA 905 Reagent Kit (Fragment Analyzer from Advanced Bioanalytical) exhibiting a sizing of 300 bp in average. Libraries were pooled and sequenced on the Illumina HiSeq 4000 (SE; 1 × 50 bp; 30–35 Mio reads/sample).

Sequence images were transformed with Illumina software BaseCaller to BCL files, which was demultiplexed to fastq files with bcl2fastq v2.17.1.14. The quality check was done using FastQC (version 0.11.5, Babraham Bioinformatics). Sequenced reads (Illumina 50 bp single-end) were mapped to the reference genome using the TopHat2 (version 2.1.0) and the quantification of transcripts was performed with Cufflinks (version 2.2.1), using Partek Flow (Partek, St. Louis, MO, USA). The data were RMA normalized. The list of differentially expressed genes (DEG) was obtained by ANOVA filtering with FDR correcting with a cut-off of adjusted *P*-value < 0.05. The fold changes are represented as log2FC (base-2 logarithm of the fold change) [14].

### 2.9 Rapamycin levels in blood

Whole-blood samples were collected during the light cycle in EDTA tubes by puncture in the facial vein of animals at 3 months of age. 50 μl of each sample was mixed with 75 μl of 0.1 M ZnSO_4_ and 125 μl of methanol during 30 min. After centrifugation at 14,000 rpm during 10 min, the supernatants were collected and injected into a liquid chromatography system H-Class (WatersCorporation) coupled to a Xevo TQS detector of mass spectrometer (MS/MS) with an electrospray ionization (Waters Corporation). The analytical separation column was a BEH C4, 1.7 μm, 2.1 × 50 mm column (Waters, Spain). The mobile phase consisted of (A) 10 mM ammonium formate and (B) methanol. A binary step gradient at a flow rate of 0.400 ml/min was employed. Up to 4 min, the ratio of A and B was kept at 50:50 (*v*/v) and from 4 min to 8.5 min the ratio was switched to 0:100 (v/v), before returning to the starting conditions [A:B, 50:50 (v/v)] up to 8.6 min [15]. Source and desolvation temperatures were set at 140 and 500 °C, respectively. Nitrogen was used as both cone gas (150 l/h) and desolvation gas (800 l/h), and argon was used as collision gas (0.14 ml/min). For detection and quantification of rapamycin, we chose the ammonium ion, which give a peak at give peak at *m*/*z* 931.6509, and the ammonium ion adducts corresponding to m/z 864.4501 and 83.4604. The standard curve of rapamycin was made at 25, 50, 100 and 200 ng/ml. The results were expressed in ng rapamycin/ml of blood.

### 2.10 Statistical analysis

Number of animals in each group were calculated in order to detect gross ~60% changes in the biomarkers measurements (based upon alpha = 0.05 and power of beta = 0.8). We used the application available in: http://www.biomath.info/power/index.htm. Animals were genotyped and randomly assigned in experimental groups in separate cages by the technician of the animal facility. Data are expressed as the mean ± SD of 5–10 experiments per group, except in the survival analysis and rotarod test, where a higher number of animals were used [[12], [13], [14], [15], [16], [17], [18], [19], [20], [21], [22], [23], [24], [25], [26], [27], [28], [29], [30], [31], [32], [33], [34], [35], [36], [37]]. A one-way ANOVA with a Tukey's post hoc test was used to compare the differences between three experimental groups. Studies with two experimental groups were evaluated using unpaired Student's *t*-test. A *P*-value of <.05 was considered to be statistically significant. Survival curve was analyzed by log-rank (Mantel-Cox) and the Gehan-Breslow-Wilcoxon tests.

### 2.11 Metabolomics analysis

The analysis of metabolites in extracts from brain and liver was performed according to the Agilent METLIN/PCDL method using a 1290 infinity UHPLC coupled to a 6550 ESI-QTOF, as previously described [36].

### 2.12 Data availability

RNA-seq data have been deposited in the ArrayExpress database at EMBL-EBI (www.ebi.ac.uk/arrayexpress) under accession number E-MTAB-7794

## 3 Results

### 3.1 Rapamycin does not modify the phenotypic features of *Coq9*^*R239X*^ mice

**Fig. 1:**
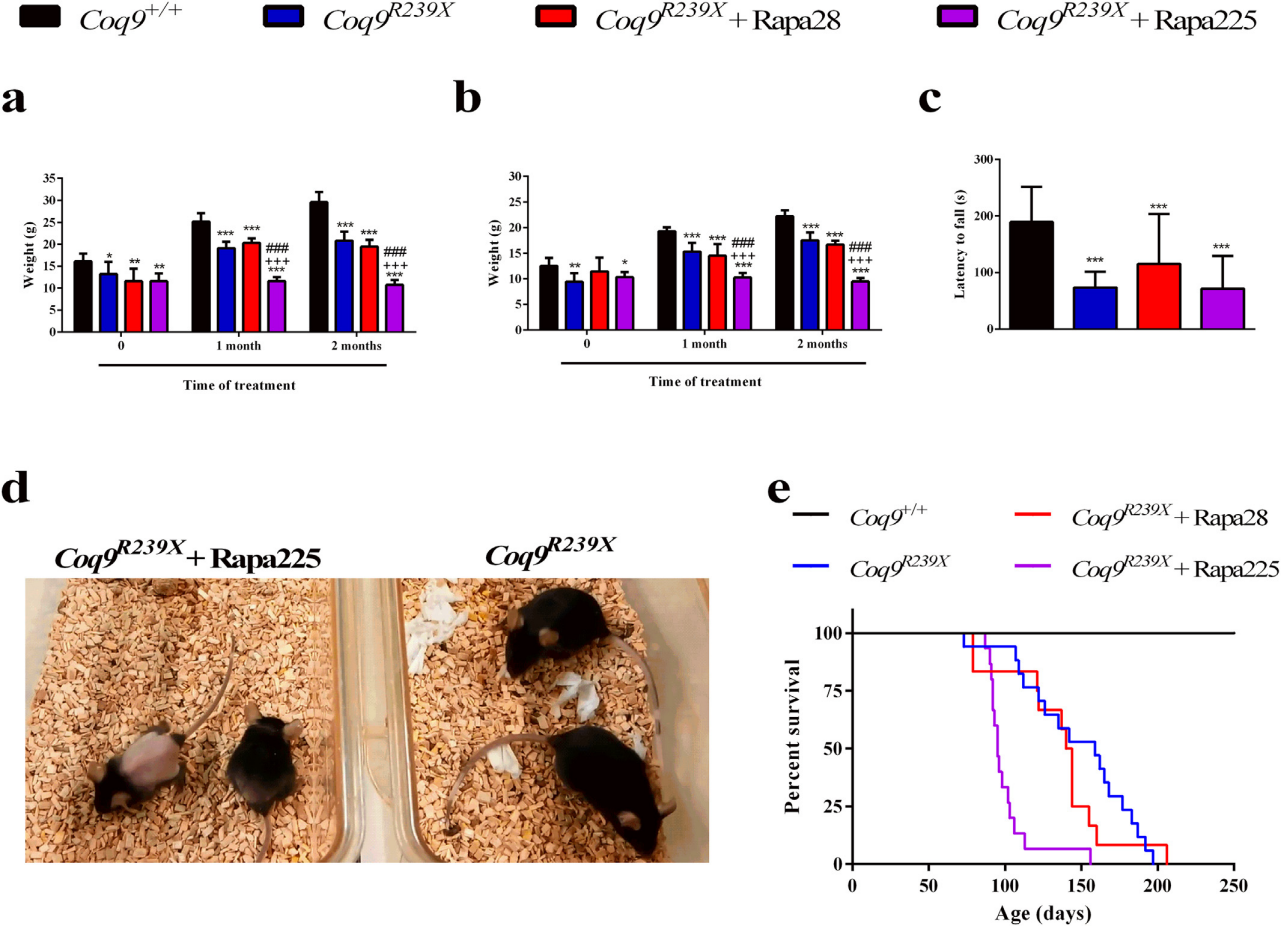
Phenotypic characterization and survival of *Coq9*^*R239X*^ mice after rapamycin treatments. (a, b) Body weight of males (a) and females (b) of *Coq9*^*+/+*^ mice, *Coq9*^*R239X*^ mice, and *Coq9*^*R239X*^ mice after 28 or 225 ppm rapamycin treatments [(a): *Coq9*^*+/+*^, *n* = 10; *Coq9*^*R239X*^, *n* = 10; *Coq9*^*R239X*^ after 28 ppm rapamycin treatment, *n* = 4; *Coq9*^*R239X*^ after 225 ppm rapamycin treatment, *n* = 10. (b): *Coq9*^*+/+*^, *n* = 10; *Coq9*^*R239X*^, *n* = 10; *Coq9*^*R239X*^ after 28 ppm rapamycin treatment, *n* = 5; *Coq9*^*R239X*^ after 225 ppm rapamycin treatment, *n* = 10]. (c) Rotarod test on *Coq9*^*+/+*^ mice (*n* = 49), *Coq9*^*R239X*^ mice (*n* = 37), and *Coq9*^*R239X*^ mice after 28 (*n* = 12) or 225 (*n* = 22) ppm rapamycin treatments. (d) Comparative image of *Coq9*^*R239X*^ mice and *Coq9*^*R239X*^ mice after 225 ppm rapamycin treatment at 3 months of age. (e) Survival curve of the *Coq9*^*+/+*^ mice, *Coq9*^*R239X*^ mice, and *Coq9*^*R239X*^ mice after 28 or 225 ppm rapamycin treatments. The treatments started at 1 month of age. *Coq9*^*+/+*^, *n* = 10, *Coq9*^*R239X*^, *n* = 17, *Coq9*^*R239X*^ mice after 28 ppm rapamycin treatment, *n* = 12, and *Coq9*^*R239X*^ mice after 225 ppm rapamycin treatment, *n* = 15. Analysis using the log-rank (Mantel-Cox) and the Gehan–Breslow–Wilcoxon tests shows significant differences between *Coq9*^*+/+*^ mice and *Coq9*^*R239X*^ mice (*P* < 0.0001 and *P* < 0.0001, respectively); *Coq9*^*+/+*^ mice and *Coq9*^*R239X*^ mice treated with rapamycin 28 ppm (*P* < 0.0001 and *P* < 0.0001, respectively); *Coq9*^*+/+*^ mice and *Coq9*^*R239X*^ mice treated with rapamycin 225 ppm (*P* < 0.0001 and *P* < 0.0001, respectively); *Coq9*^*R239X*^ mice and *Coq9*^*R239X*^ mice treated with rapamycin 225 ppm (*P* < 0.0001 and *P* < 0.0001, respectively); and *Coq9*^*R239X*^ mice treated with rapamycin 28 ppm and *Coq9*^*R239X*^ mice treated with rapamycin 225 ppm (*P* = .0032 and *P* = .0090, respectively). Data information: Data are expressed as mean ± SD. ^⁎^*P* < 0.05; ^⁎⁎^*P* < 0.01; ^⁎⁎⁎^*P* < 0.001; *Coq9*^*+/+*^ versus *Coq9*^*R239X*^ or *Coq9*^*R239X*^ after 28 or 225 ppm rapamycin treatments. ^+++^*P* < 0.001; *Coq9*^*R239X*^ versus *Coq9*^*R239X*^ after 28 or 225 ppm rapamycin treatments. ^###^*P* < 0.001; *Coq9*^*R239X*^ after 28 ppm versus *Coq9*^*R239X*^ after 225 ppm rapamycin treatment (one-way ANOVA with a Tukey's post hoc test or *t*-test).

To test whether rapamycin may have general therapeutic effects in mitochondrial encephalomyopathies, we treated *Coq9*^*R239X*^ mice with two different concentrations of active encapsulated rapamycin added into the chow. The two concentrations were 28 ppm (low dose), which has been used in aging studies [2]; and 225 ppm (high dose), which is similar to the dose used in the *Ndufs4*^*−/−*^ mouse model [16]. The treatments started at the first month of age, previous to the emergence of the severe disease symptoms. *Coq9*^*R239X*^ showed a reduced size and body weight, compared to the *Coq9*^*+/+*^ mice, in both males (Fig. 1a) and females (Fig. 1b). These differences were already present at the first month of age and became more evident over the time. Low dose of rapamycin did not induce any change on the body weight in males and females of *Coq9*^*R239X*^ mice compared to untreated *Coq9*^*R239X*^ mice, while high-dose of rapamycin induced a dramatic decrease in the body weight (Fig. 1a, b and d). *Coq9*^*R239X*^ mice also show impaired motor coordination, tested by rotarod assay. Rapamycin, at either low or high dose, was not able to increase the latency to fall of *Coq9*^*R239X*^ mice on the rotarod assay (Fig. 1c). Consistent with these data, rapamycin therapy did not extend the survival of *Coq9*^*R239X*^ mice. Even, the lifespan was significantly shortened at the higher dose of rapamycin, for which most of the animal died between 3 and 4 months of age and all the mice were dead before 6 months of age (Fig. 1e).

### 3.2 Brain injuries are still observed in *Coq9*^*R239X*^ mice treated with rapamycin

**Fig. 2:**
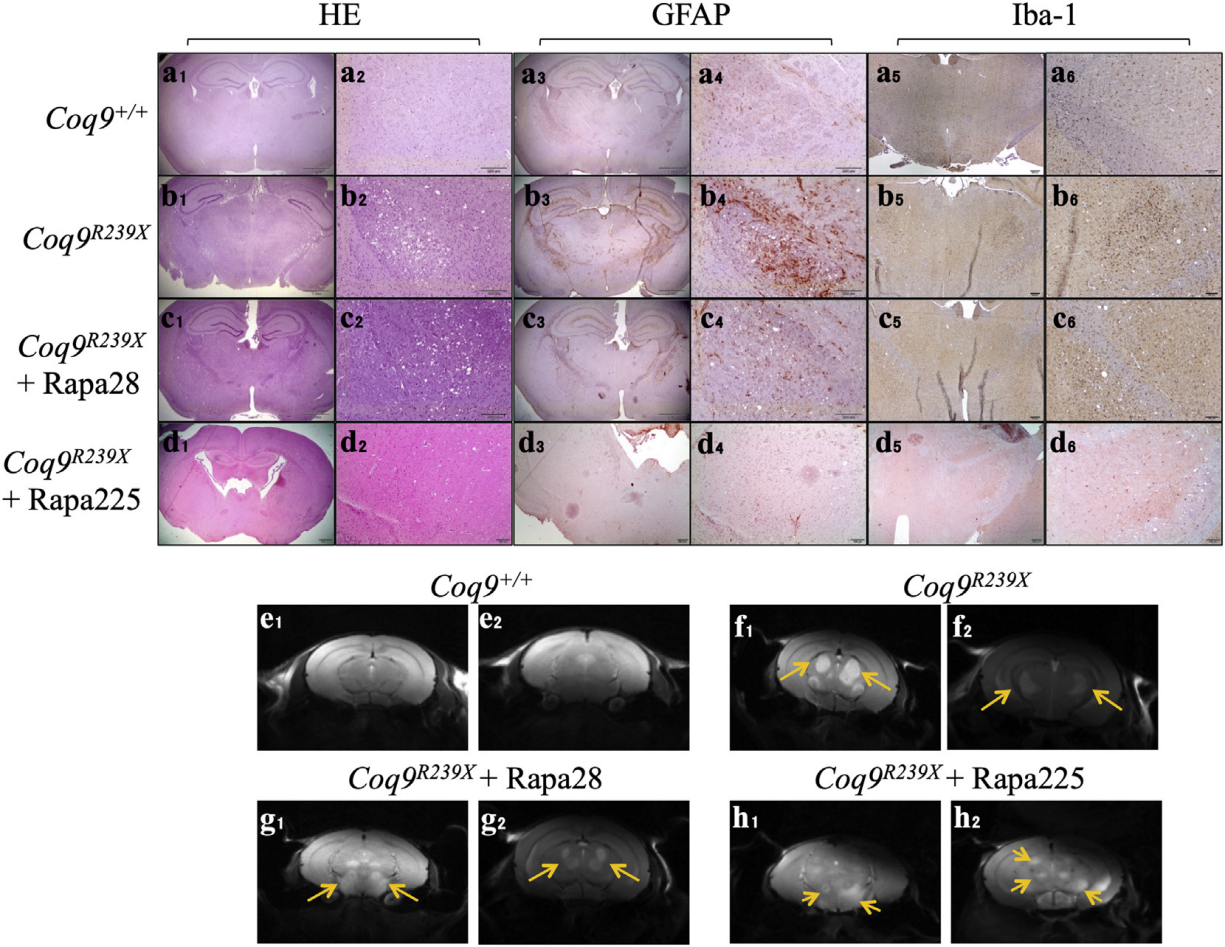
Pathological features in brain tissue sections of *Coq9*^*R239X*^ after 28 or 225 ppm rapamycin treatments. (a_1_–a_2_ to d_1_–d_2_) Hematoxylin and eosin stain in the diencephalon of *Coq9*^*+/+*^ mice (a_1_ and a_2_), *Coq9*^*R239X*^ mice (b_1_ and b_2_), *Coq9*^*R239X*^ mice after 28 ppm rapamycin treatment (c_1_ and c_2_), and *Coq9*^*R239X*^ mice after 225 ppm rapamycin treatment (d_1_ and d_2_). (a_3_-a_4_ to d_3_-d_4_) Anti-GFAP stain in the diencephalon of *Coq9*^*+/+*^ mice (a_3_ and a_4_), *Coq9*^*R239X*^ mice (b_3_ and b_4_), *Coq9*^*R239X*^ mice after 28 ppm rapamycin treatment (c_3_ and c_4_), and *Coq9*^*R239X*^ mice after 225 ppm rapamycin treatment (d_3_ and d_4_). (a_5_-a_6_ to d_5_-d_6_) Anti-Iba-1 stain in the diencephalon of *Coq9*^*+/+*^ mice (a_5_ and a_6_), *Coq9*^*R239X*^ mice (b_5_ and b_6_), *Coq9*^*R239X*^ mice after 28 ppm rapamycin treatment (c_5_ and c_6_), and *Coq9*^*R239X*^ mice after 225 ppm rapamycin treatment (d_5_ and d_6_). (e_1_–e_2_ to h_1_-h_2_) Magnetic Resonance Images of the diencephalon of *Coq9*^*+/+*^ mice (e_1_ and e_2_)*, Coq9*^*R239X*^ mice (f_1_ and f_2_), and *Coq9*^*R239X*^ mice after 28 (g_1_ and g_2_) or 225 ppm rapamycin treatment (h_1_ and h_2_). Scale bars: 500 μm (a_1_–d_1_); 200 μm (a_2_–d_2_); 500 μm (a_3_–d_3_); 200 μm (a_4_–d_4_); 500 μm (a_5_–d_5_); 200 μm (a_6_–d_6_). The yellow arrows indicated the areas of increased T2 signal, which is characteristic of lesions in specific brain areas. (For interpretation of the references to colour in this figure legend, the reader is referred to the web version of this article.)

The lack of phenotypic improvement after rapamycin therapy should be also observed at histopathologic level, since we previously reported that *Coq9*^*R239X*^ mice show severe spongiosis and reactive astrogliosis in different brain areas, such as the pons, the *medulla oblongata* and the diencephalon. Thus, we observed a profound vacuolization and proliferation of astrocytes (as indicates the astrocytes' marker GFAP) on the diencephalon and the pons of *Coq9*^*R239X*^ mice (Fig. 2, b1–b4; Fig. S1; Fig. S2, b1–b4; Fig. S3), compared to *Coq9*^*+/+*^ mice (Fig. 2, a1–a4; Fig. S1; Fig. S2 a1–a4; Fig. S3). These histopathologic changes were also apparent in the treated *Coq9*^*R239X*^ mice, although the astrogliosis was partially reduced, especially in the diencephalon (Fig. 2, c1–c4, d1–d4; Fig. S1; Fig. S2, c1–c4, d1–d4; Fig. S3). Because rapamycin has potent immunosuppressive properties and it induced a reduction of microgliosis in the *Ndufs4*^*−/−*^ mice [1], we also tried to detect the microglia in the cerebral preparations. However, microgliosis was not generally observed in the diencephalon and the pons of *Coq9*^*R239X*^ mice (Fig. 2, b5–b6; Fig. S1; Fig. S2, b5–b6) and only some small spots, very focused and infrequent, of microglia proliferation were observed in some preparations. The Iba-1 images of these cerebral areas in the mutant mice under rapamycin treatment, either at low (Fig. 2, c5–c6; Fig. S1; Fig. S2, c5–c6) or high doses (Fig. 2, d5–d6; Fig. S1; Fig. S2, d5–d6), were comparable to those on *Coq9*^*+/+*^ (Fig. 2, a5–a6; Fig. S1, a5–a6) and *Coq9*^*R239X*^ mice (Fig. 2, b5–b6; Fig. S1; Fig. S2, b5-b6).

The pathological manifestations in the brain of *Coq9*^*R239X*^ mice were further corroborated by magnetic resonance imaging, in which *Coq9*^*R239X*^ mice showed abundant lesions (Fig. 2, f1–f2) that were persistent in the brain of the treated mice at either low or high doses of rapamycin (Fig. 2, g1–h2). Therefore, rapamycin treatment did not correct the main pathological features on the brain of *Coq9*^*R239X*^ mice and, consequently, it did not improve the phenotype and it did not increase the lifespan on this mouse model.

### 3.3 Rapamycin inhibits mTORC1 but it does not induce autophagy in *Coq9*^*R239X*^ mice

**Fig. 3:**
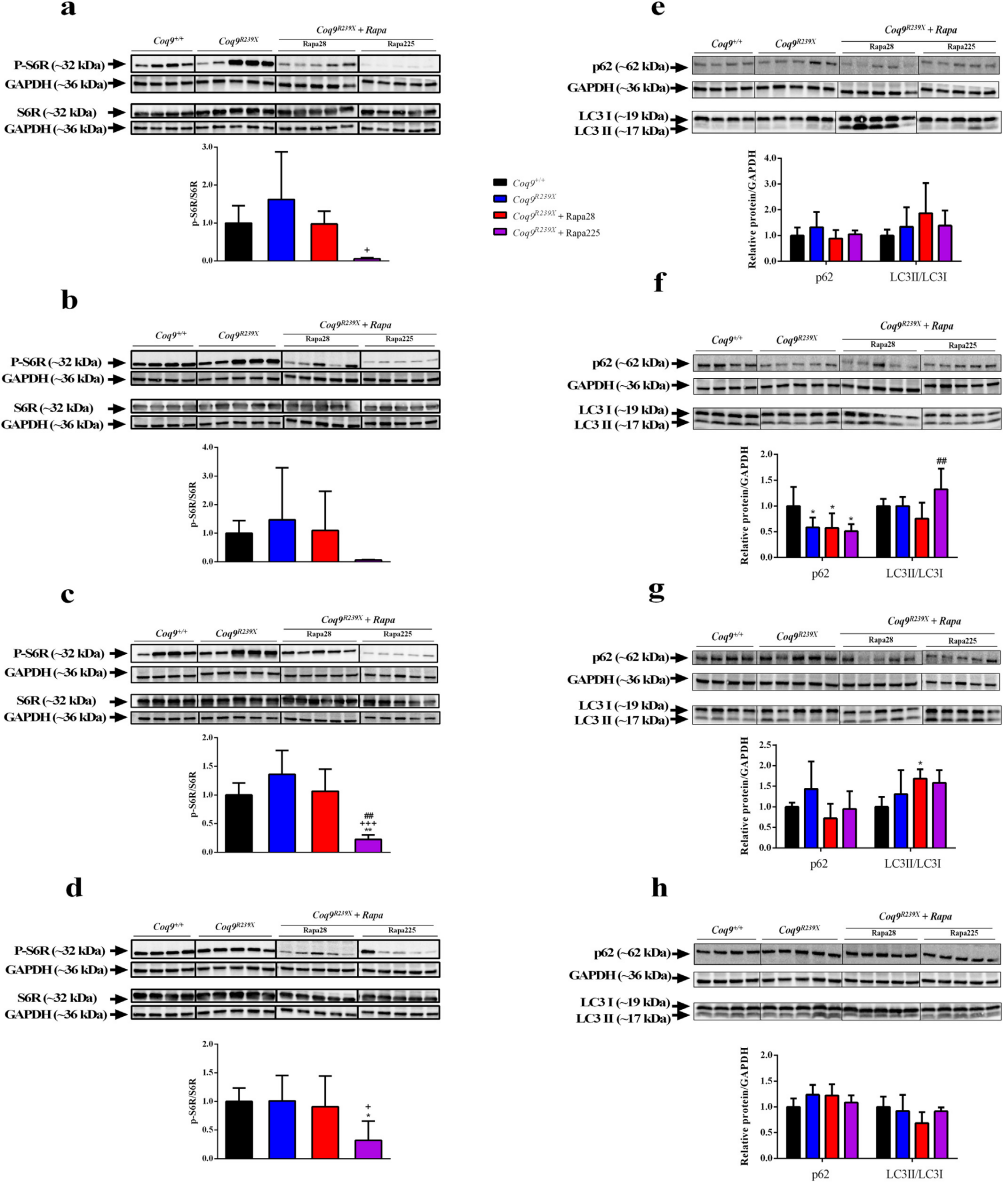
mTORC1 inhibition and autophagy alterations in *Coq9*^*R239X*^ tissues after rapamycin treatments. (a–d) Representative western blots of the phosphorylation state of the mTORC1 target S6R protein in heart (a), kidney (b), liver (c), and brain (d). (e–h) Representative images of western blots of p62 and LC3 autophagy markers in heart (e), kidney (f), liver (g), and brain (h). *Coq9*^*+/+*^, *n* = 4–8; *Coq9*^*R239X*^, *n* = 5–10; *Coq9*^*R239X*^ mice after 28 ppm rapamycin treatment, *n* = 5; and *Coq9*^*R239X*^ mice after 225 ppm rapamycin treatment, *n* = 5. Data are expressed as mean ± SD. ^⁎^*P* < 0.05; ^⁎⁎^*P* < 0.01; *Coq9*^*+/+*^ versus *Coq9*^*R239X*^ or *Coq9*^*R239X*^ after 28 or 225 ppm rapamycin treatment. ^+^*P* < 0.05; ^+++^*P* < 0.001; *Coq9*^*R239X*^ versus *Coq9*^*R239X*^ after 28 or 225 ppm rapamycin treatment; ^##^*P* < 0.01; *Coq9*^*R239X*^ after 28 ppm versus *Coq9*^*R239X*^ after 225 ppm rapamycin treatment (one-way ANOVA with a Tukey's post hoc test or *t*-test).

The levels of rapamycin in the blood increased during its oral administration (Table 1), and its levels in mutant mice treated with rapamycin at 225 ppm were comparable to those published elsewhere [16]. To determine if the supplementation of rapamycin in the chow was able to inhibit mTORC1 in the *Coq9*^*R239X*^ mice, we analyzed the phosphorylation state of S6R protein (p-S6R), one of the targets of mTORC1, in heart, kidney, liver and brain. The p-S6R/S6R ratio was similar in the four tissues of *Coq9*^*+/+*^ and *Coq9*^*R239X*^ mice (Fig. 3a–d), although the tissues of *Coq9*^*R239X*^ mice showed a high variability in the values. At the lower dose, rapamycin did not significantly modify the p-S6R/S6R ratio in any of the four tissues (Fig. 3a–d). At the higher dose, however, all the examined tissues responded to rapamycin inhibition, as they experienced a reduction in the p-S6R/S6R ratio, compared to *Coq9*^*+/+*^ and *Coq9*^*R239X*^ mice (Fig. 3a–d).

**Table 1:** Levels of rapamycin in blood.

Genotype	Administration route	Number of samples with detected levels of rapamycin	Type of samples	Levels of rapamycin (ng/ml)
*Coq9*^*+/+*^	not treated	0/5	Blood	UND
*Coq9*^*R239X*^	not treated	0/5	Blood	UND
*Coq9*^*R239X*^	Oral (chow at 225 ppm)	9/9	Blood	605.3 ± 436.2

Rapamaycin therapy started in animals at 1 month of age and the blood samples were collected in animals at three moths of age.

One of the mTORC1 downstream target process is autophagy. Particularly, several studies have shown that the inhibition of mTORC1 induces the mechanisms to promote autophagy in mammalian cells [17]. Thus, we have analyzed two different markers of autophagy, the LC3II/LC3I ratio and the levels of p62, in our experimental conditions. In the heart and the brain, we did not detect any difference of these autophagy markers between the four experimental groups (Fig. 3e and h). In the kidneys, the levels of p62 were decreased in the three experimental groups of *Coq9*^*R239X*^ mice, while the LC3II/I ratio was only increased in *Coq9*^*R239X*^ mice treated with the high dose of rapamycin (Fig. 3f), suggesting that this therapeutic condition may exacerbate the autophagic process. In the liver, we only observed an increase of the LC3II/I ratio in *Coq9*^*R239X*^ mice treated with the high dose of rapamycin, compared to *Coq9*^*+/+*^ mice (Fig. 3g). Therefore, contrary to what it was expected, there was no evidence of general autophagy induction in *Coq9*^*R239X*^ mice after 2 months of rapamycin treatment, a fact that may be attributed to an early autophagy response induced by CoQ deficiency, as it has been previously reported in vitro [18] and it is also suggested in our analysis in the brain at 2 months of age (Fig. S4).

### 3.4 Rapamycin modifies the transcriptomics and metabolomics profiles in *Coq9*^*R239X*^ mice

Although the low dose of rapamycin does not induce a widespread inhibition of mTORC1, its systemic effects can encourage profound alterations in different cellular and tissue pathways, as it has previously reported [19]. To check this possibility, we performed a transcriptomics and metabolomics analyses in the brain of *Coq9*^*R239X*^ mice treated with the low dose of rapamycin. To better understand the changes induced by rapamycin, those analyses were extended to a transcriptomics analysis in the brain of *Coq9*^*R239X*^ mice treated with the high dose of rapamycin; and to a metabolomics analysis in the liver of *Coq9*^*R239X*^ mice treated with the low dose of rapamycin since this tissue is a primary target of the circulating rapamycin [19].

**Fig. 4:**
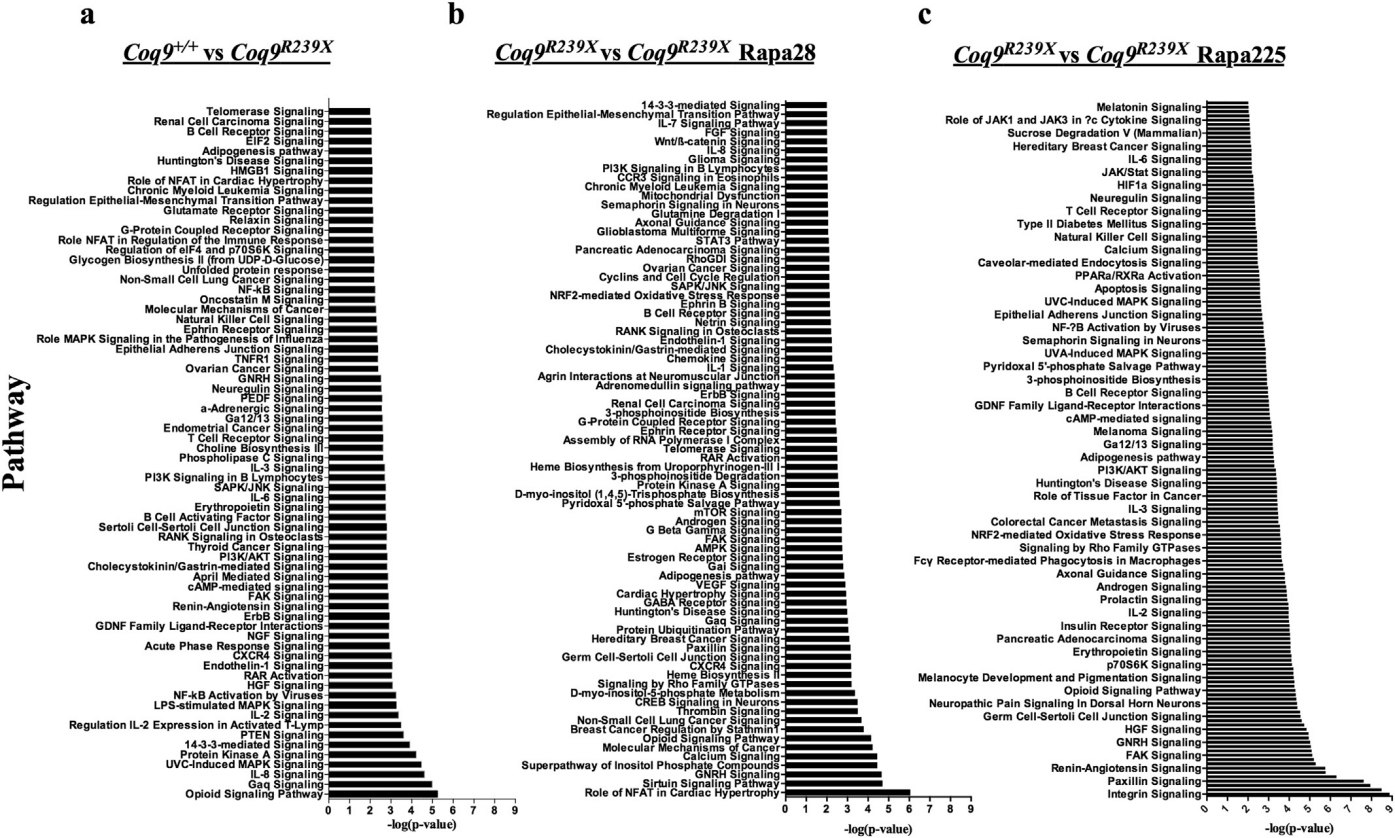
RNA-Seq analysis using the IPA software. Effect of rapamycin on the biological pathways in midbrain of *Coq9*^*R239X*^ mice. Only pathways with –log (*P*-value) ≥ 2 with a maximum of 70 of the most significant pathways are considered for representation. Comparison of *Coq9*^*+/+*^ and *Coq9*^*R239X*^ mice (a), and *Coq9*^*R239X*^ mice and *Coq9*^*R239X*^ mice after 28 (b) or 225 (c) ppm rapamycin treatments biological processes. *Coq9*^*+/+*^, *n* = 7; *Coq9*^*R239X*^, *n* = 8; *Coq9*^*R239X*^ mice after 28 ppm rapamycin treatment, *n* = 5; and *Coq9*^*R239X*^ mice after 225 ppm rapamycin treatment, *n* = 4.

Our transcriptomics analysis revealed 138 differentially expressed genes (DEGs) between *Coq9*^*R239X*^ and *Coq9*^*+/+*^ mice (82 up-regulated and 56 down-regulated) (Supplementary File 1); 2876 DEGs between *Coq9*^*R239X*^ mice and *Coq9*^*R239X*^ mice treated with the low dose of rapamycin (166 up-regulated and 2710 down-regulated) (Supplementary File 1); and 3007 DEGs between *Coq9*^*R239X*^ mice and *Coq9*^*R239X*^ mice treated with the high dose of rapamycin (2919 up-regulated and 88 down-regulated) (Supplementary File 1) (adjusted *P* value < 0.05). The data analysis with the Ingenuity Pathway Analysis (IPA) software gave us the identification of altered pathways and networks (Fig. 4). Among others, we observed an alteration in pathways related with inflammation (e.g. IL-8, MAPK, IL-2, NF-κB or immune cells receptors) in the midbrain of *Coq9*^*R239X*^ mice compared to *Coq9*^*+/+*^ mice (Fig. 4a). Most of these pathways were still altered after the treatment with rapamycin, both at low and high doses (Fig. 4b and c). Nevertheless, we observed that rapamycin treatment altered some pathways related to mitochondrial biology, such as, mTOR signaling, sirtuin signaling pathway, AMPK signaling, NRF-2 mediated oxidative stress response, PPARα/RXRα signaling or mitochondrial dysfunction (Fig. 4b and c; Fig. S5), all of them potential therapeutic targets in mitochondrial diseases [[20], [21], [22], [23]].

**Fig. 5:**
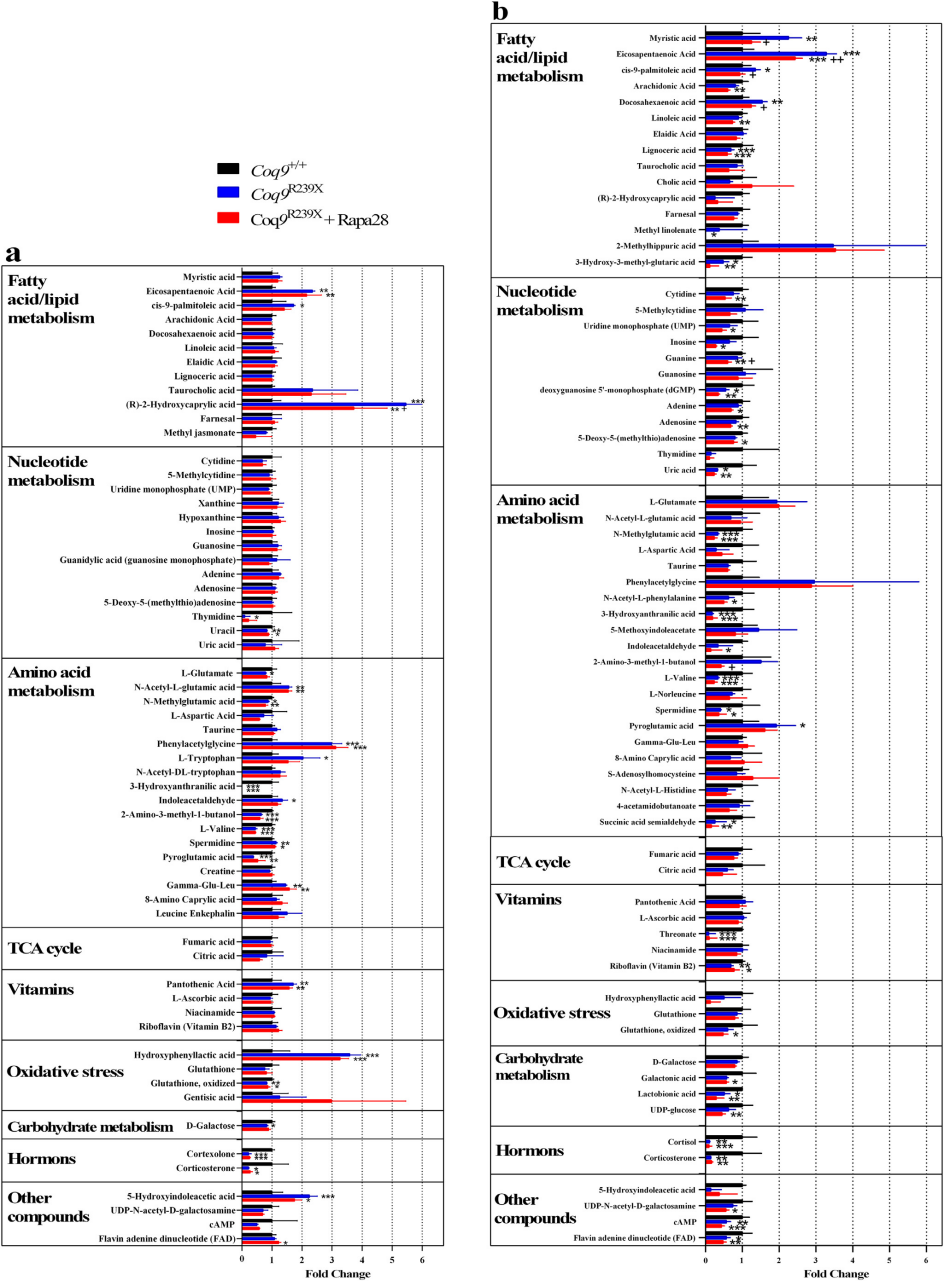
Metabolomics analysis. Analysis of brain (a) and liver (b) metabolites of *Coq9*^*+/+*^ (black bars), *Coq9*^*R239X*^ (blue bars), and *Coq9*^*R239X*^ after 28 ppm rapamycin treatment (red bars) mice (*n* = 4 for each group). Data are expressed as mean ± SD. ^⁎^*P* < 0.05; ^⁎⁎^*P* < 0.01; ^⁎⁎⁎^*P* < 0.001; *Coq9*^*+/+*^ versus *Coq9*^*R239X*^ or *Coq9*^*R239X*^ after 28 ppm rapamycin treatment. ^+^*P* < 0.05; ^++^*P* < 0.01; *Coq9*^*R239X*^ versus *Coq9*^*R239X*^ after 28 ppm rapamycin treatment (one-way ANOVA with a Tukey's post hoc test or *t*-test). (For interpretation of the references to colour in this figure legend, the reader is referred to the web version of this article.)

The alterations in gene expression were reflected in changes in the metabolic profile in the experimental groups. The main pathways affected by the mutation and/or by the treatment with low dose of rapamycin were related to lipid metabolism, nucleotides metabolism, amino acid metabolism, vitamins, oxidative stress, carbohydrates metabolism and corticosterone (Fig. 5). Those changes were more pronounced in the liver (Fig. 5b) than in the brain (Fig. 5a); and especially relevant were the effects of low dose of rapamycin on the nucleotides metabolism and the lipid metabolism in liver (Fig. 5b).

### 3.5 Rapamycin treatment does not interfere in the mitochondrial bioenergetics of *Coq9*^*R239X*^ mice

**Fig. 6:**
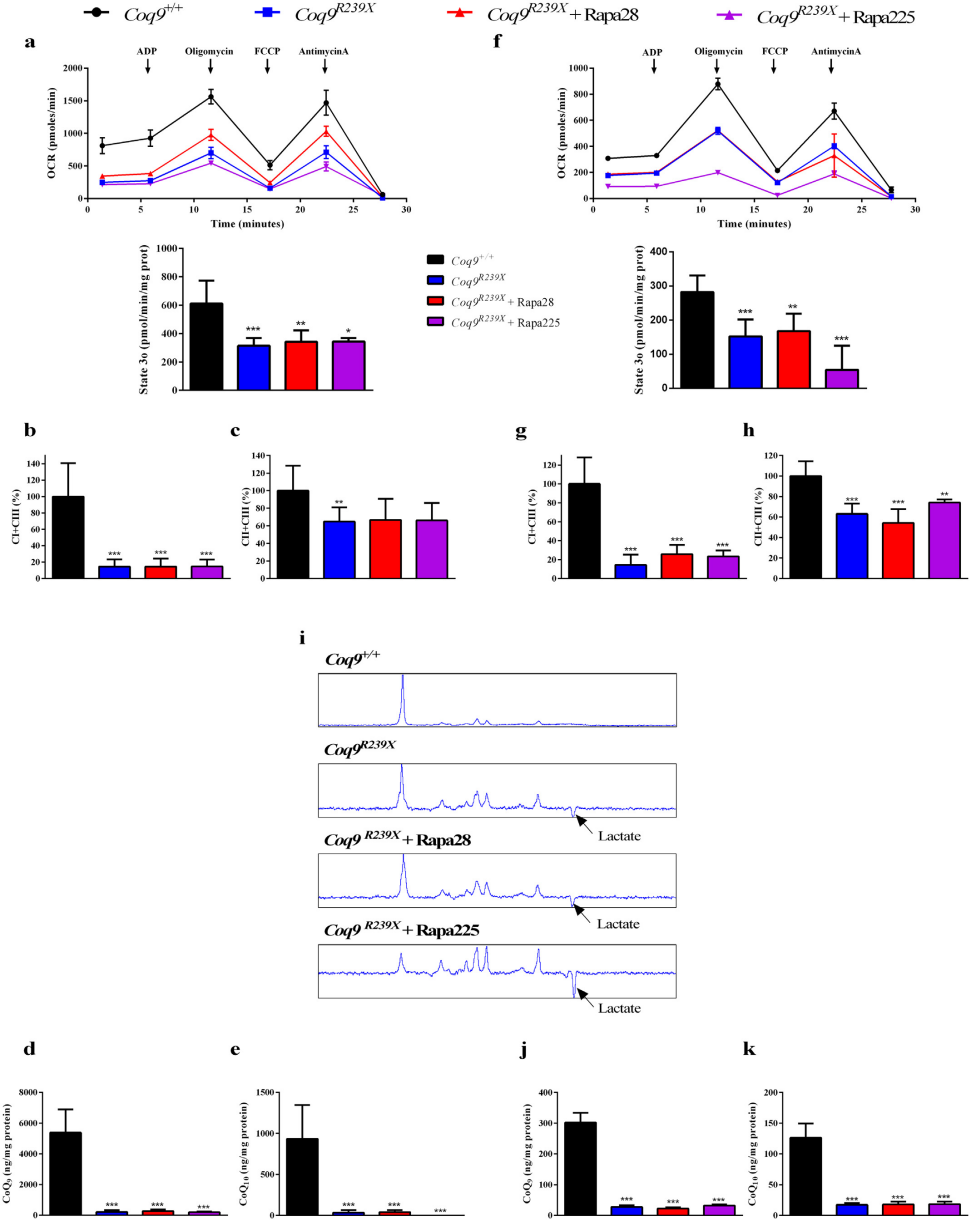
Mitochondrial bioenergetics is not affected after rapamycin treatments. (a,f) Oxygen consumption rate (top) and ADP-stimulated respiration (State 3o, bottom) in the presence of ADP and substrates measured by mitochondrial respiration in kidney (a) and brain (f). (a) *Coq9*^*+/+*^, *n* = 3; *Coq9*^*R239X*^, *n* = 3; *Coq9*^*R239X*^ mice after 28 ppm rapamycin treatment, *n* = 3; and *Coq9*^*R239X*^ mice after 225 ppm rapamycin treatment, *n* = 4. (f): *Coq9*^*+/+*^, *n* = 3; *Coq9*^*R239X*^, *n* = 3; *Coq9*^*R239X*^ mice after 28 ppm rapamycin treatment, *n* = 4; and *Coq9*^*R239X*^ mice after 225 ppm rapamycin treatment, *n* = 4. (b,g) CoQ-dependent Complex I + III activity in kidney (b) and brain (g). (b) *Coq9*^*+/+*^, *n* = 12; *Coq9*^*R239X*^, *n* = 11; *Coq9*^*R239X*^ mice after 28 ppm rapamycin treatment, *n* = 4; and *Coq9*^*R239X*^ mice after 225 ppm rapamycin treatment, *n* = 4. (g) *Coq9*^*+/+*^, *n* = 8; *Coq9*^*R239X*^, *n* = 9; *Coq9*^*R239X*^ mice after 28 ppm rapamycin treatment, *n* = 5; and *Coq9*^*R239X*^ mice after 225 ppm rapamycin treatment, *n* = 4. (c, h) CoQ-dependent Complex II + III activity in kidney (c) and brain (h). (c) *Coq9*^*+/+*^, *n* = 13; *Coq9*^*R239X*^, *n* = 12; *Coq9*^*R239X*^ mice after 28 ppm rapamycin treatment, *n* = 4; and *Coq9*^*R239X*^ mice after 225 ppm rapamycin treatment, *n* = 4. (h) *Coq9*^*+/+*^, *n* = 9; *Coq9*^*R239X*^, *n* = 9; *Coq9*^*R239X*^ mice after 28 ppm rapamycin treatment, *n* = 5; and *Coq9*^*R239X*^ mice after 225 ppm rapamycin treatment, *n* = 4. (d, e, j, k) Levels of CoQ_9_ and CoQ_10_ in kidney (d and e, respectively) and brain (j and k, respectively). (d, j) *Coq9*^*+/+*^, *n* = 9; *Coq9*^*R239X*^, *n* = 9; *Coq9*^*R239X*^ mice after 28 ppm rapamycin treatment, *n* = 5; and *Coq9*^*R239X*^ mice after 225 ppm rapamycin treatment, *n* = 5. (e, k) *Coq9*^*+/+*^, *n* = 8; *Coq9*^*R239X*^, *n* = 9; *Coq9*^*R239X*^ mice after 28 ppm rapamycin treatment, *n* = 6; and *Coq9*^*R239X*^ mice after 225 ppm rapamycin treatment, *n* = 5 (i) Lactate peak in the brain of *Coq9*^+/+^ mice, *Coq9*^*R239X*^ mice and *Coq9*^*R239X*^ mice after 28 ppm. Data are expressed as mean ± SD. ^⁎^*P* < 0.05; ^⁎⁎^*P* < 0.01; ^⁎⁎⁎^*P* < 0.001; *Coq9*^*+/+*^ versus *Coq9*^*R239X*^ or *Coq9*^*R239X*^ after 28 or 225 ppm rapamycin treatment (one-way ANOVA with a Tukey's post hoc test or *t*-test).

To check whether the transcriptomics and metabolomics alterations could influence the mitochondrial bioenergetics, we first measured the mitochondrial respiration in the kidneys and the brain, two of the prevalent affected tissues in primary CoQ deficiency. Both tissues of *Coq9*^*R239X*^ mice showed an impaired mitochondrial respiration, with lower phosphorylating respiratory state (state 3o) compared to the same tissues in *Coq9*^*+/+*^ mice (Fig. 6a and f). After 2 months of rapamycin treatment, at either low or high dose, the mitochondrial respiration in the kidneys and the brain of *Coq9*^*R239X*^ mice was also lower than in the same tissues in *Coq9*^*+/+*^ mice (Fig. 6a and f). In agreement with that, the activities of the mitochondrial CI + CIII and CII + CIII were decreased in the kidneys and the brain of *Coq9*^*R239X*^ mice, and the rapamycin treatment did not induce any change on these parameters (Fig. 6b, c, g and h). The reduction on the OXPHOS capacity induces the anaerobic metabolism, as it is reflected in the detection of lactate in the spectroscopy of the brain of *Coq9*^*R239X*^ mice (Fig. 5i). The rapamycin treatment did not normalize the energy metabolism and accumulation of lactate was also detected in the mutant animals treated with rapamycin (Fig. 6i). Together, these data may indicate that rapamycin did no act in OXPHOS capacity, including the levels of CoQ, which were reduced in the three experimental groups of *Coq9*^*R239X*^ mice compared to *Coq9*^*+/+*^ mice (Fig. 6d, e, j and k). Consistent with that, the accumulation of DMQ, the substrate of the hydroxylase COQ7, persisted in the *Coq9*^*R239X*^ mice treated with either low or high dose of rapamycin (Fig. S6).

### 3.6 Modulation of mTORC1 downstream pathways did not provide therapeutic benefits in *Coq9*^*R239X*^ mice

Because the inhibition of mTORC1 produce pleiotropic results and induce side effects that can mask its therapeutic potential, we also tested the treatment with two molecules that can modulate specific mTORC1 downstream pathways, i.e.: trehalose, that can induce autophagy in an mTOR-independent manner and it has been considered as a neuroprotector in some neurodegenerative diseases [5]; and PF-4708671, that inhibits the protein and lipid synthesis by the specific inhibition of the S6K1 protein [6]. The treatment with trehalose did not induce any morphological improvement in the pons of the *Coq9*^*R239X*^ mice (Fig. S7, a5–a6, b5–b6), since the spongiosis and astrogliosis were similar to those in the untreated mutant animals (Fig. S7, a3–a4, b3–b4). Those features were absent in the wild-type animals (Fig. S7, a1–a2, b1–b2). The CoQ-dependent mitochondrial complexes activities, CI + III and CII + CIII, were reduced in *Coq9*^*R239X*^ mice and *Coq9*^*R239X*^ mice treated with trehalose, compared to *Coq9*^*+/+*^ mice, in both kidneys (Fig. S7c and e) and brain (Fig. S7d and f). Also, the spongiosis and astrogliosis persisted in the pons of the *Coq9*^*R239X*^ mice during the treatment with PF-4708671 (Fig. S7, g5–g6, h5–h6), as it was clearly observable after comparing the images to those of the untreated *Coq9*^*R239X*^ mice (Fig. S7, g3–g4, h3–h4), and *Coq9*^*+/+*^ mice (Fig. S7, g1–g2, h1–h2). The immunohistochemistry for the detection of microglia did not detect significant and reliable differences between the three experimental groups (Fig. S7, i1–i6). Consistent with these results, the mitochondrial respiration was decreased in *Coq9*^*R239X*^ mice and *Coq9*^*R239X*^ mice treated with PF-4708671, compared to *Coq9*^*+/+*^ mice, in both kidneys (Fig. S7j and k) and brain (Fig. S7l and m). Because the two treatments did not show any improvement in the histopathological features and the bioenergetic status of the *Coq9*^*R239X*^ mice (Fig. S7), both treatments failed to increase the survival of the mutant animals (data not shown).

## 4 Discussion

It has been reported that the inhibition of mTORC1 by rapamycin administration has therapeutic effects, at different intensities, in a variety of preclinical models of mitochondrial diseases with diverse molecular defects and phenotypes. However, our study fails to demonstrate that the inhibition of mTORC1 induces any therapeutic outcomes in a mouse model of mitochondrial encephalopathy due to CoQ deficiency. Therefore, contrary to what it could be speculated from the previous studies, this work points out that chronic rapamycin treatment is not a valid therapy for all cases of mitochondrial diseases, not even for all cases of mitochondrial encephalopathies where the central nervous system is the most affected structure.

In 2013, Johnson and colleagues showed that intraperitoneal (i.p.) administration of high doses of rapamycin dramatically reduced the gliosis and the histopathological signs on a mouse model of Leigh syndrome due to mitochondrial complex I deficiency (*Ndufs4*^*−/−*^ model), thus increasing the survival [1]. This effect of i.p. administration of high doses of rapamycin was independently corroborated [24], and two other studies reported that oral administration of different doses of rapamycin, starting from 42 ppm of dietary administration, delayed the development of neurological symptoms and extended the lifespan in *Ndufs4*^*−/−*^ mice, confirming also that the route of administration is not a limitation [16,25]. However, in the four studies the underlying mitochondrial dysfunction was not mitigated, and the mechanism by which rapamycin produced the therapeutic outcomes was not sufficiently elucidated. Nevertheless, theses studies opened a new perspective for testing rapamycin therapy in other cases of mitochondrial diseases. Thus, in other mouse model of mtDNA depletion and encephalopathy due to thymidine kinase 2 (TK2) deficiency (the *Tk2*^*H126N*^ model), low doses of oral rapamycin induced a very mild increase in lifespan without any improvement in the morphological features [19]; and in two mouse models with milder phenotypes, the rapamycin administration also resulted in therapeutic benefits, i.e.: (1) in the mouse model of mitochondrial myopathy with COX deficiency due to the muscle-specific ablation of the *Cox15* gene (the *Cox15*^*sm/sm*^ model), the i.p. administration of high doses of rapamycin improved the locomotor activity, corrected the histopathological features and increased the COX activity in muscle [26]; and (2) in the mouse model of late-onset mitochondrial myopathy due to a defect in the helicase Twinkle (the *Twnk*^*dup*^ model), the i.p. administration of high doses of rapamycin downregulated the components of the integrated mitochondrial stress response, thus reversing the myopathy progression [27]. Contrary to these results, the oral administration of rapamycin, either at low (28 ppm) or high dose (225 ppm), did not produce any therapeutic benefits in *Coq9*^*R239X*^ mice. Even, the group of animals with the high dose of rapamycin experienced an aggravation on the brain lesions, resulting in a mild decrease in the survival, which could be attributed to the reported side effects of this drug, e.g. thrombocytopenia and hyperlipidemia, impaired wound healing, nephrotoxicity, and altered insulin sensitivity [28]. In this regard, it is important to note that inhibition of mTORC1 was only clearly detected at the high dose, although the low dose induced a very mild inhibition only in heart and liver. This is consistent with the fact that rapamycin is able to induce morphological and phenotypic improvements with survival increase in *Ndufs4*^*−/−*^ mice in a dose-dependent manner [1,16,25]. In fact, our transcriptomics analysis in midbrain revealed dose-dependent differences in the DEGs, which may account systemic effects of the rapamycin administration and specific effects related to the inhibition of mTORC1 in the midbrain.

The differences in the therapeutic effects of rapamycin administration in diverse mouse models of mitochondrial diseases must be explained by the specific therapeutic mechanisms induced by rapamycin. First, in the symptomatic tissues of *Ndufs4*^*−/−*^ and *Twnk*^*dup*^ mice, the p-S6/S6 ratio, used as a marker of the mTORC1 activity, was significantly increased compared to age match wild-type animals, while rapamycin treatment reduced this p-S6/S6 ratio [1,27]. In the *Coq9*^*R239X*^ mice, we observed the same effect of rapamycin at the high dose but the brain of both untreated mutant mice and wild-type mice have similar p-S6/S6 ratio. Similarly, an overactivation of mTORC1 was not observed in *Tk2*^*H126N*^ and *Cox15*^*sm/sm*^ mice, although in those models rapamycin was therapeutically relevant [19,26]. Because of the inhibition of mTORC1, rapamycin was able to lessen the microgliosis and downregulate microglia-related inflammatory genes in *Ndufs4*^*−/−*^ mice [1,24]. This effect could not be induced in *Coq9*^*R239X*^ mice because we were not able to detect any consistent sign of microgliosis in this mouse model. Moreover, the differentially-expressed inflammatory genes observed in the transcriptomics analysis were related to the proliferation of astrocytes and not to the proliferation of microglia [9] and some of the key inflammatory genes downregulated by rapamycin in *Ndufs4*^*−/−*^ mice (*Tlr2*, *Cxcl10*, *Ccl5* and *Aif1*) were not picked up in our transcriptomics analysis [24]. Another therapeutic mechanism attributed to rapamycin in mitochondrial diseases is the activation of autophagy and the lysosomal biogenesis [17,26]. Our transcriptomics analysis did not show any clue of lysosomal biogenesis induction and the levels of autophagy markers did not suggest a general rapamycin-mediated autophagy induction. The lack of a strong autophagic activity may be due to that fact that CoQ deficiency induces an early autophagy response, as it is suggested in the brain *of Coq9*^*R239X*^ at 2 months of age, and as it has been reported in vitro [18]. This early event may be disrupted over the time by an impairment of lysosomes induced by the oxidative damage [29]. In fact, CoQ deficiency induces oxidative stress in vitro and in vivo [[30], [31], [32]], and the pons and diencephalon of *Coq9*^*R239X*^ mice show increased signals of markers of oxidative damage [4,9,11]. A third rapamycin-mediated therapeutic mechanism is the inhibition of the integrated mitochondrial stress response, which includes the repression of the mitochondrial unfolded protein response (UPRmt). However, the deleterious effect of the induction of UPRmt has been only identified in the model *Twnk*^*dup*^ of mitochondrial myopathy [27,33], and the levels of two proteins involved in UPRmt, the mitochondrial proteins stress-70 protein (HSPA9 or GRP75) and ATP-dependent Clp protease proteolytic subunit (CLPP), are unaltered in other mouse model of CoQ deficiency due to a different mutation in the *Coq9* gene [34]. The last rapamycin-mediated therapeutic mechanism is the partial bypass of the OXPHOS defect by tapping into alternative energy reserves, such as amino acids and lipids [19]. This mechanism could be therapeutically relevant in cases of deficiencies in mitochondrial Complex I or Complex II. Nevertheless, this effect may have some limitations under CoQ deficiency because different pathways of the energy metabolism, e.g. carbohydrate metabolism, lipid metabolism, sulfide metabolism and pyrimidine metabolism, converge in the CoQ-junction to enter electrons into the mitochondrial respiratory chain [35,36]. Moreover, we observed some changes in the levels of metabolites related to amino acid, carbohydrate, nucleotides and lipid metabolism in liver, with a limited effect on the brain, as it was reported in the *Tk2*^*H126N*^ model [19]; but the changes in lipid metabolism were related to eicosanoid metabolism, rather than to β-oxidation and energy metabolism.

While our results suggest that rapamycin administration is not a therapeutic option in primary CoQ deficiency, a recent study in the mouse model of CoQ deficiency due to a point mutation in the CoQ biosynthetic gene *Pdss2* (the *Pdss2*^*kd/kd*^ model) showed that oral administration of high doses of rapamycin (225 ppm) reduced the albuminuria in this mouse model of nephrotic syndrome [37]. However, the survival was not analyzed and the morphological structure of the renal glomeruli was not shown [37]. Thus, the reduction in the levels of the albumin in the urine could merely reflect the reduction in the phosphorylation of 4E-BP1 and S6K1 because of the inhibition of mTORC1 and, consequently, a perturbation of the protein synthesis [17]. Also, we need to take into account that the different response to rapamycin administration may be due to two important dissimilarities between the *Pdss2*^*kd/kd*^ and *Coq9*^*R239X*^ models: 1) *Pdss2*^*kd/kd*^ mice have severe CoQ deficiency while *Coq9*^*R239X*^ mice have severe CoQ deficiency together with an accumulation of the toxic intermediate demethoxyubiquinone (DMQ) [9], which is still accumulated under rapamycin treatment and produces a non-functional competition with CoQ [9,38,39]; and 2) while *Pdss2*^*kd/kd*^ mice develop nephrotic syndrome and die between 5 and 11 months age [23], *Coq9*^*R239X*^ mice develop a mitochondrial encephalopathy, with a potential contribution of endocrine interactions in the pathological features of the diseases [9], and die between 3 and 7 months age [4,9].

In conclusion, our study suggests that the use of rapamycin in patients with mitochondrial diseases must be evaluated for each particular case. The fail of therapeutic benefits in the *Coq9*^*R239X*^ mouse model may be due to different factors, such as: 1) the lack of microgliosis-derived neuroinflammation in this model of encephalopathy; 2) the lysosomes impairment, which avoid the induction of an autophagy flux; or 3) the need of some residual function of the CoQ-junction in order to promote the supply of electron into the mitochondrial respiratory chain from different metabolic sources. Therefore, the translation of this therapy into the clinic requires additional studies and the consideration of the particularities of each mitochondrial disease. Moreover, the development of rational therapies must provide better therapeutic outcomes with minimum side-effects, as it has been demonstrated, at preclinical level, with the dNTPs/nucleosides therapy in Tk2 deficiency [40,41], with AICAR administration or OPA1 overexpression in COX deficiency [20,42], or with the β-resorcylic acid therapy in cases of CoQ deficiency due to defects in the CoQ biosynthetic genes *Coq7* or *Coq9* [9,43].

## Appendix A Supplementary data

Supplementary material 1 Image 1

Supplementary material 2 Image 2
