# Supplementary material for: Rapamycin administration is not a valid therapeutic strategy for every case of mitochondrial disease
Source: eBioMedicine. 2019 Mar 18;42:511–23. doi: 10.1016/j.ebiom.2019.03.025 (PMC6492073; doi:10.1016/j.ebiom.2019.03.025)
Supplement: Supplementary file 1 — Supplementary material 1 [file mmc1.docx]

**SUPPLEMENTARY MATERIAL**

**Rapamycin Administration is not a Valid Therapeutic Strategy for Every Case of Mitochondrial Disease**

Eliana Barriocanal-Casado^1,2^, Agustín Hidalgo-Gutiérrez^1,2^, Nuno Raimundo^4^, Pilar González-García^1,2^, Darío Acuña-Castroviejo^1,2,3^, Germaine Escames^1,2,3^, Luis C. López^1,2,3*^

*Correspondence email: luisca@ugr.es

**SUPPLEMENTAL DATA**

**
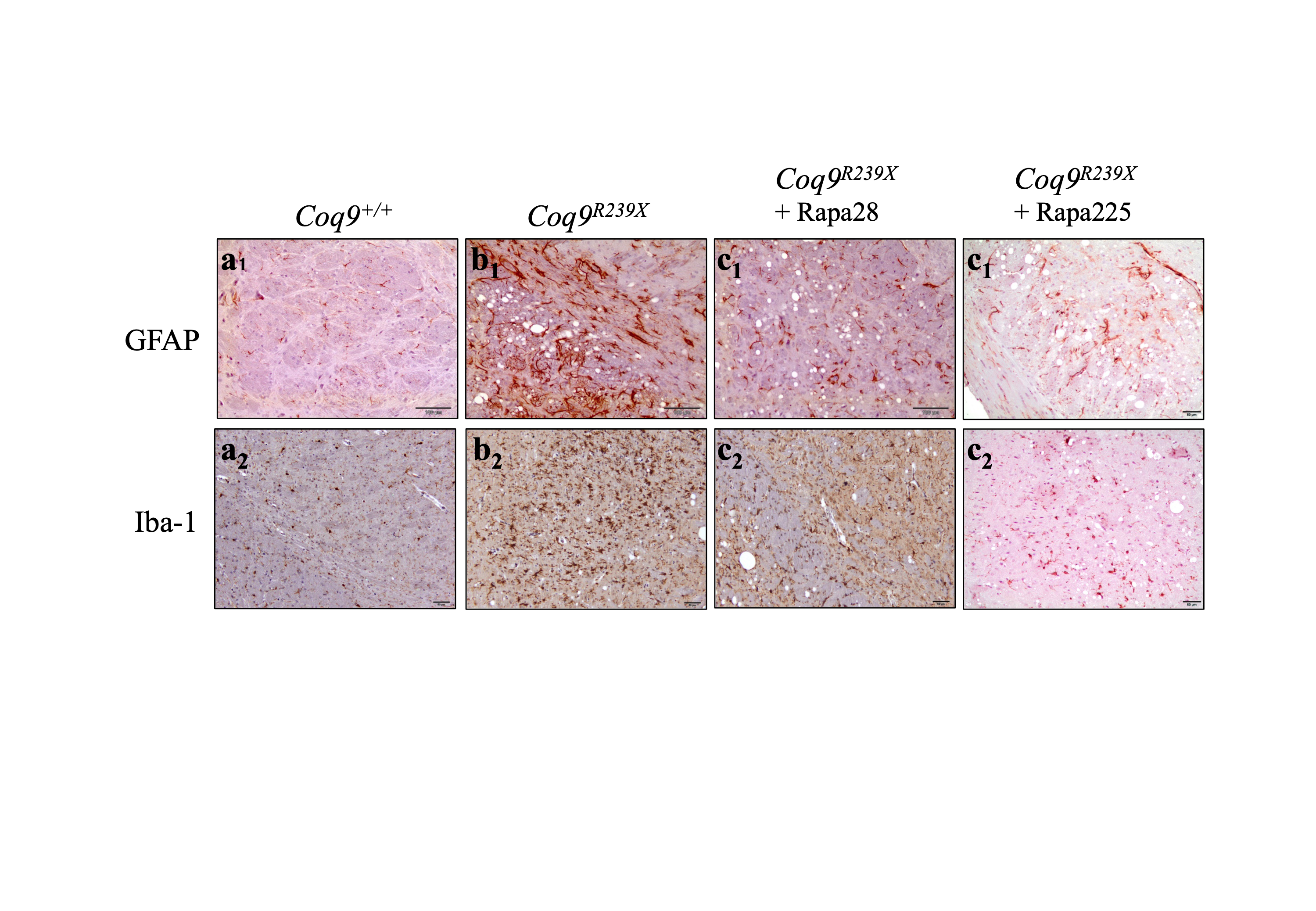
**

**Figure S1. Magnified images of the GFAP and Iba-1 shown in figure 3.**

Anti-GFAP stain in the diencephalon of *Coq9^+/+^* mice (**a_1_**), *Coq9^R239X^* mice (**b_1_**), *Coq9^R239X^* mice after 28 ppm rapamycin treatment (**c**_1_), and *Coq9^R239X^* mice after 225 ppm rapamycin treatment (**d_1_**). Anti-Iba-1 stain in the pons of *Coq9^+/+^* mice (**a_2_**), *Coq9^R239X^* mice (**b_2_**), *Coq9^R239X^* mice after 28 ppm rapamycin treatment (**c_2_**), and *Coq9^R239X^* mice after 225 ppm rapamycin treatment (**d_2_**).

**
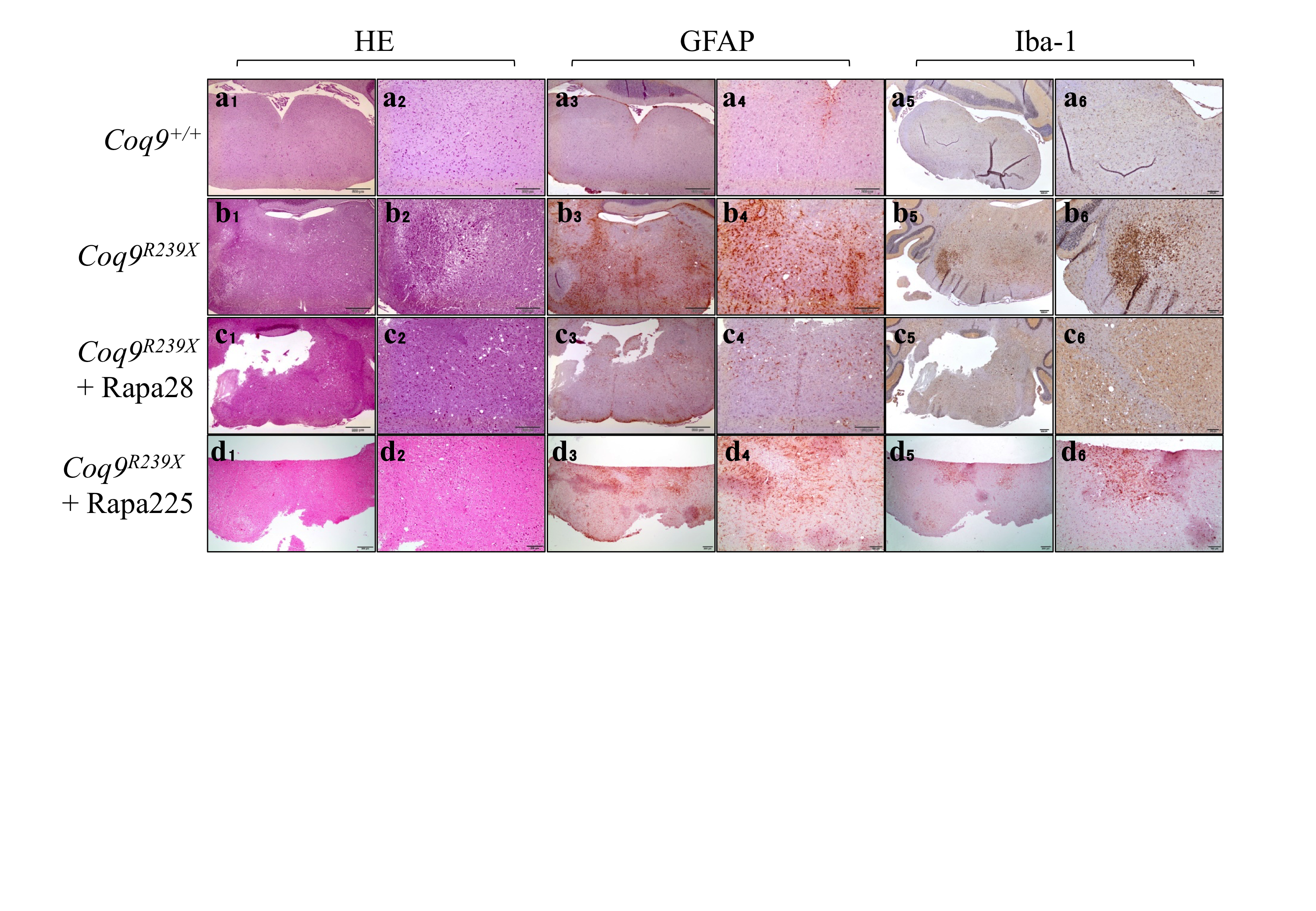
**

**Figure S2. Histopathological features in the pons of *Coq9^R239X^* after 28 or 225 ppm rapamycin treatments.**

(**a_1_-a_2_ to d_1_-d_2_**) Hematoxylin and eosin stain in the pons of *Coq9^+/+^* mice (**a_1_** and **a_2_**), *Coq9^R239X^* mice (**b_1_** and **b_2_**), *Coq9^R239X^* mice after 28 ppm rapamycin treatment (**c_1_** and **c_2_**), and *Coq9^R239X^* mice after 225 ppm rapamycin treatment (**d_1_** and **d_2_**). (**a_3_-a_4_ to d_3_-d_4_**) Anti-GFAP stain in the pons of *Coq9^+/+^* mice (**a_3_** and **a_4_**), *Coq9^R239X^* mice (**b_3_** and **b_4_**), *Coq9^R239X^* mice after 28 ppm rapamycin treatment (**c_3_** and **c_4_**), and *Coq9^R239X^* mice after 225 ppm rapamycin treatment (**d_3_** and **d_4_**). (**a_5_-a_6_ to d_5_-d_6_**) Anti-Iba-1 stain in the pons of *Coq9^+/+^* mice (**a_5_** and **a_6_**), *Coq9^R239X^* mice (**b_5_** and **b_6_**), *Coq9^R239X^* mice after 28 ppm rapamycin treatment (**c_5_** and **c_6_**), and *Coq9^R239X^* mice after 225 ppm rapamycin treatment (**d_5_** and **d_6_**).

Scale bars: 500 μm (**a_1_**–**d_1_**); 200 μm (**a_2_**–**d_2_**); 500 μm (**a_3_**–**d_3_**); 200 μm (**a_4_**–**d_4_**); 500 μm (**a_5_**–**d_5_**); 200 μm (**a_6_**–**d_6_**).

**
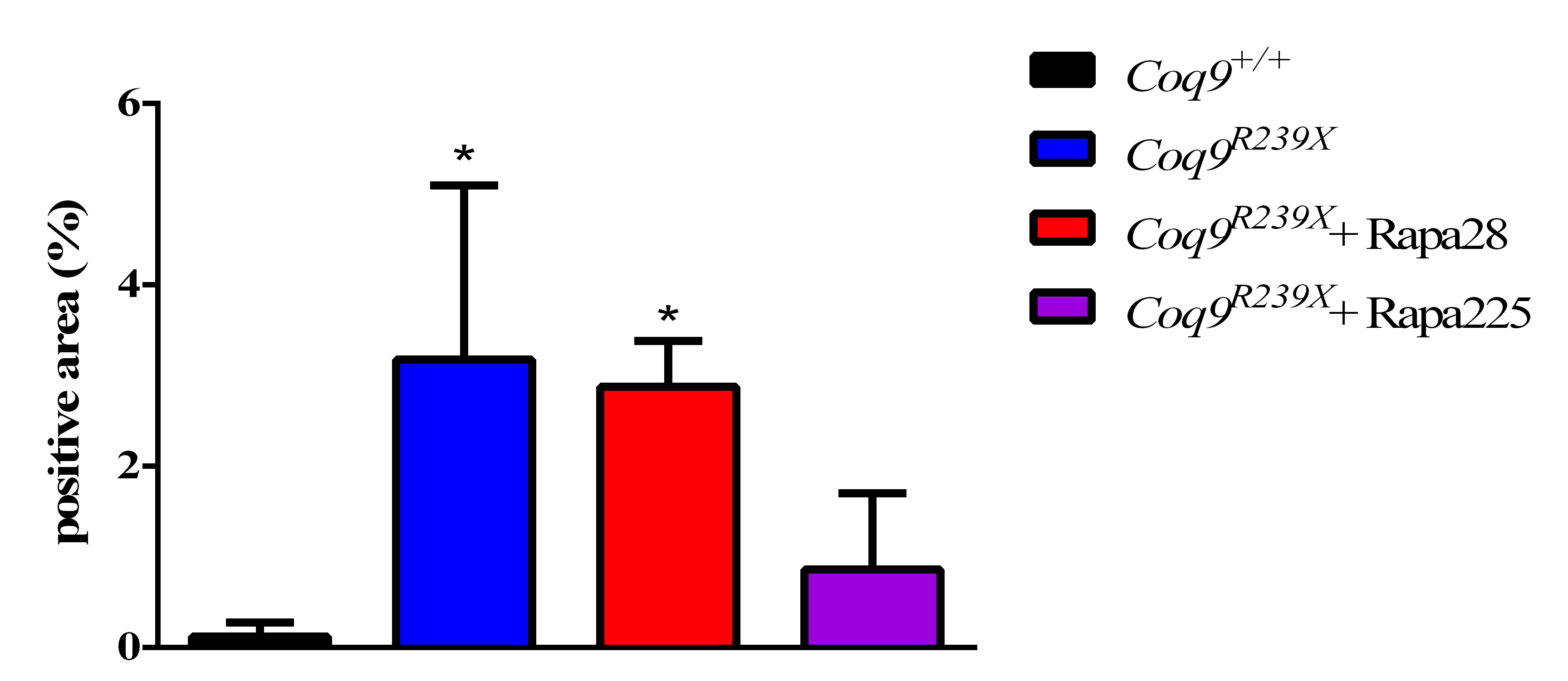
**

**Figure S3. Percentage of GFAP positive signal in the images of diencephalon.** Results correspond to the mean ± SD, as determined by the software ImageJ. Data are expressed as mean ± SD. **P* < 0.05; *Coq9^+/+^* versus *Coq9^R239X^* or *Coq9^R239X^* after 28 or 225 ppm rapamycin treatment (one-way ANOVA with a Tukey’s post hoc test).

**
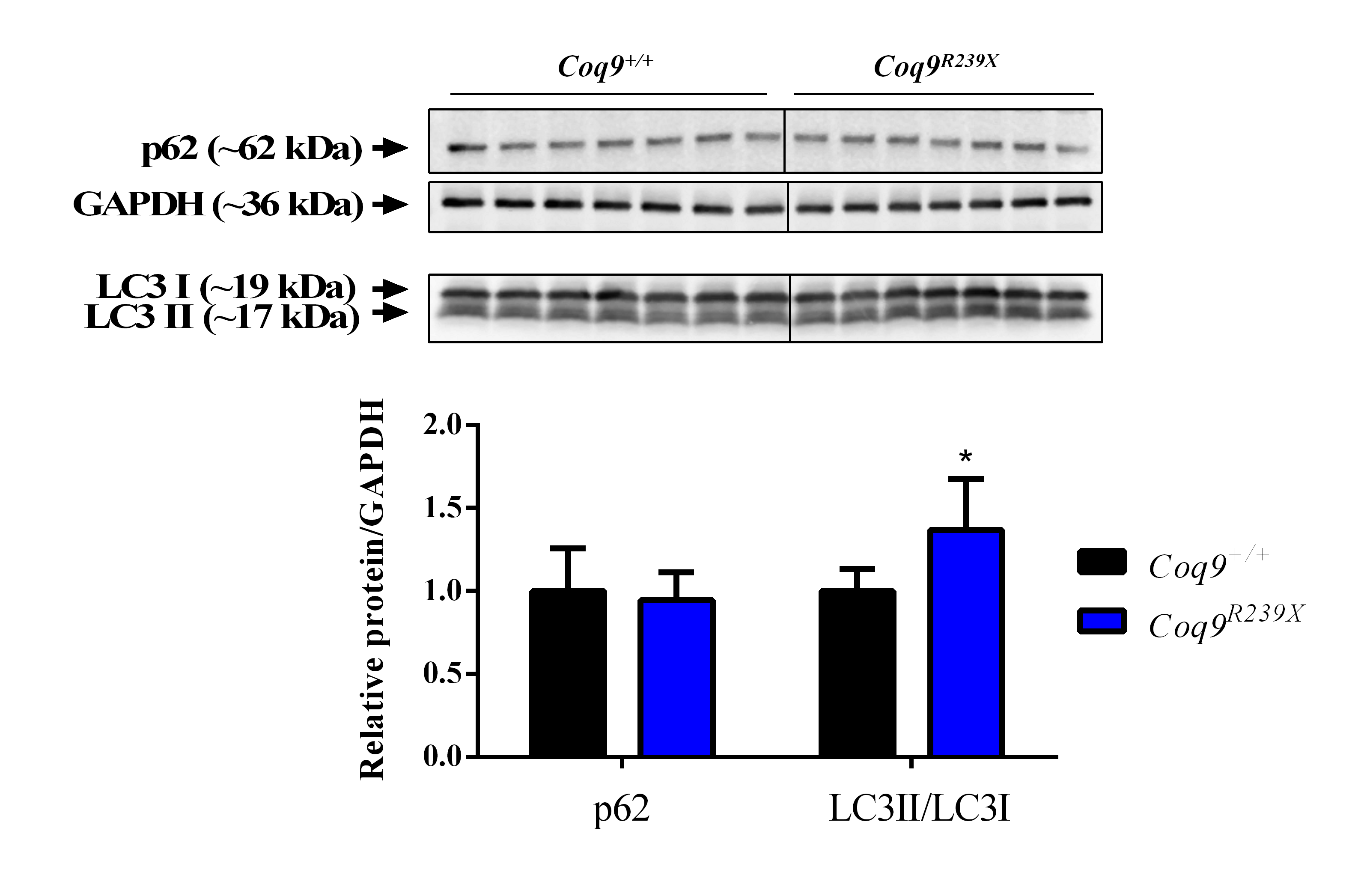
**

**Figure S4. Representative images of western blots of p62 and LC3 autophagy markers in the brain of *Coq9^+/+^* and *Coq9^R239X^* mice at 2 months of age.** Data are expressed as mean ± SD. N=7 in each experimental group. **P* < 0.05; *Coq9^+/+^* versus *Coq9^R239X^* (*t*-test).

**
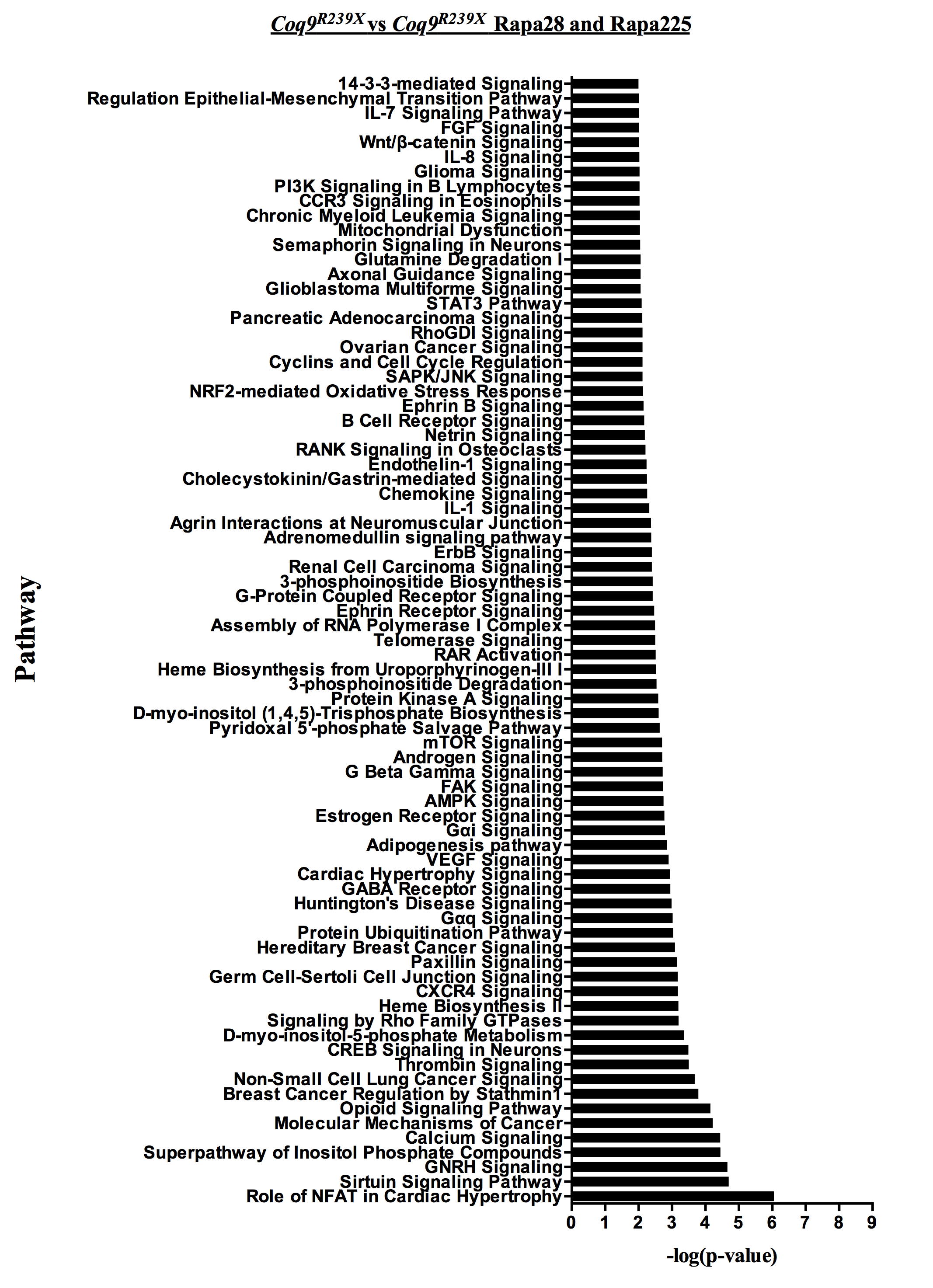
**

**Figure S5. RNA-Seq analysis using the IPA software. Common altered pathways after 28 and 225 ppm rapamycin treatments.** Only pathways with –log (p-value) ≥ 2 are considered for representation.

**
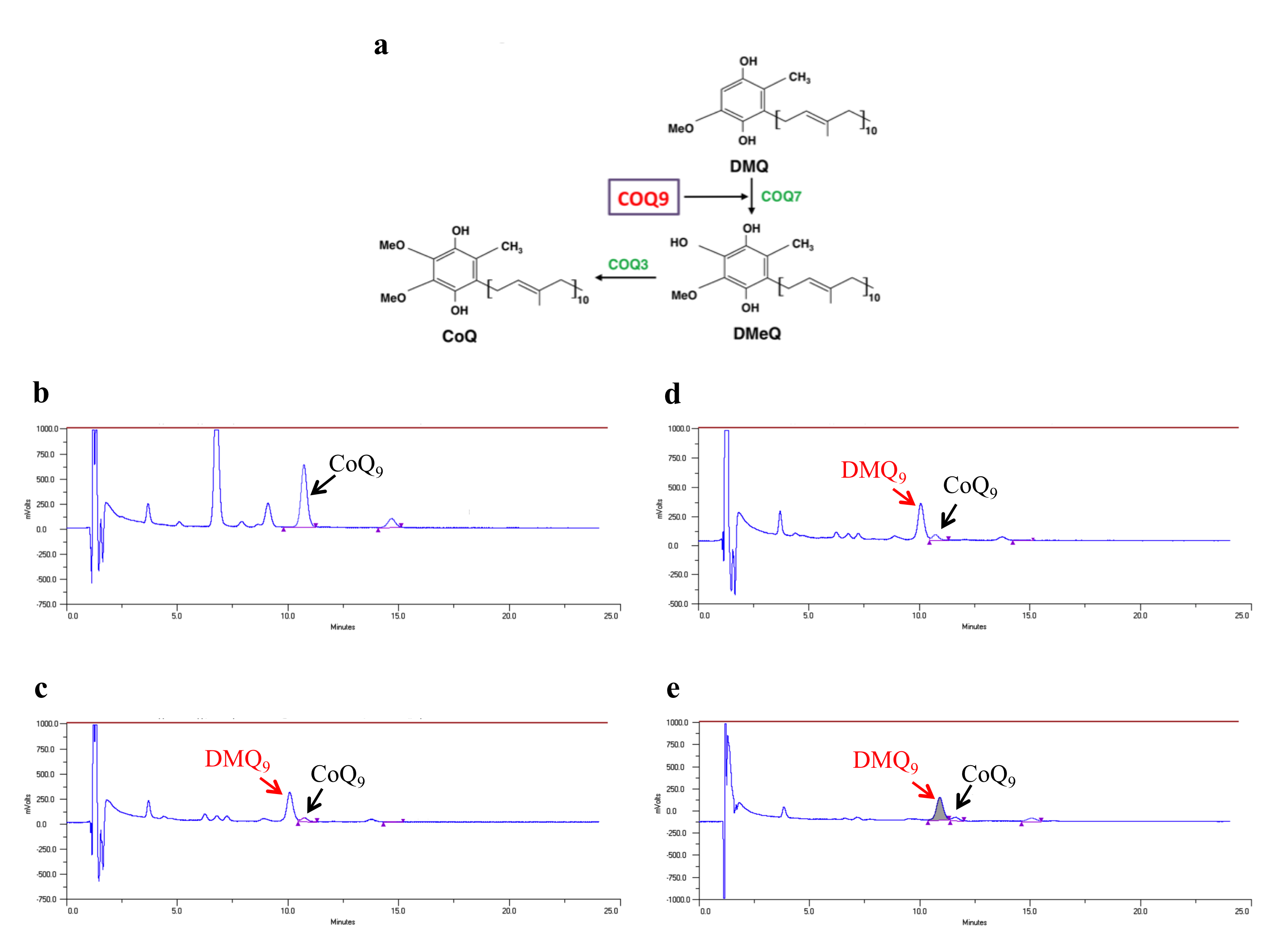
**

**Figure S6. DMQ accumulation after 28 or 225 rapamycin treatments.**

(**a**) Schematic representation of the specific step in the CoQ biosynthetic pathway in which the COQ9 protein is involved. The hydroxylase COQ7 needs COQ9 for the hydroxylation of DMQ to DMeQ, and consequently, dysfunction of COQ7 or COQ9 results in accumulation of DMQ.

(**d-e**) Representative chromatographs showing the different quinones in the kidney of *Coq9^+/+^* mice (**b**), *Coq9^R239X^* mice (**c**), *Coq9^R239X^* mice after 28 ppm rapamycin treatment (**d**), and *Coq9^R239X^* mice after 225 ppm rapamycin treatment (**e**).

**
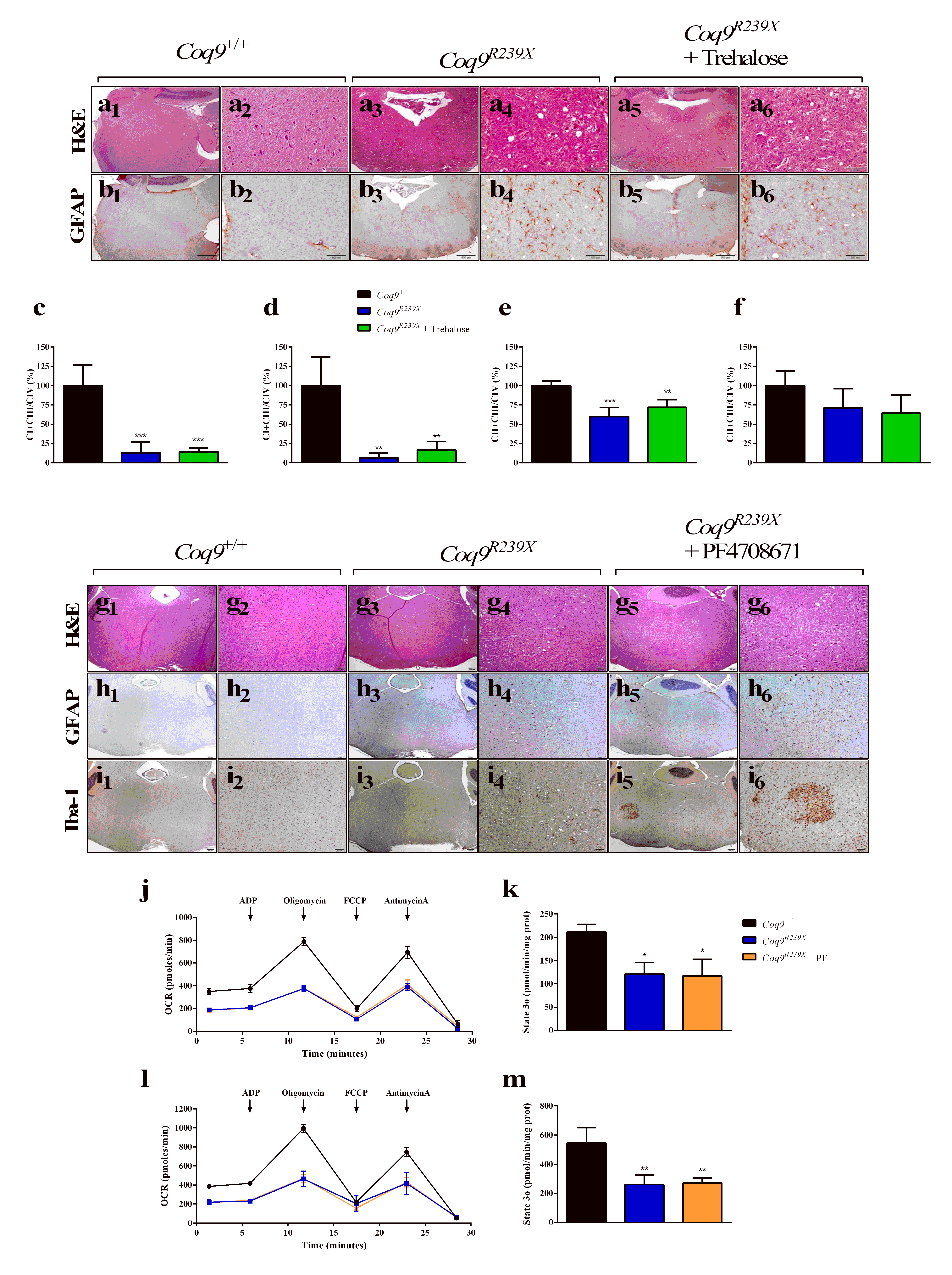
**

**Figure S7. Evaluation of other therapies for *Coq9^R239X^* mice based on the modulation of mTOR downstream pathways.**

(**a_1_** to **a_6_**) Hematoxylin and eosin stain in the pons of *Coq9^+/+^* mice (**a_1_** and **a_2_**), *Coq9^R239X^* mice (**a_3_** and **a_4_**)_,_ and *Coq9^R239X^* mice after trehalose treatment (**a_5_** and **a_6_**). (**b_1_** to **b_6_**) Anti-GFAP stain in the pons of *Coq9^+/+^* mice (**b_1_** and **b_2_**), *Coq9^R239X^* mice (**b_3_** and **b_4_**), and *Coq9^R239X^* mice after trehalose treatment (**b_5_** and **b_6_**). (**c-f**) CoQ-dependent Complex I +III and Complex II +III activities in brain (**c** and **e**, respectively) and kidney (**d** and **f**, respectively). (**g_1_** to **g_6_** ) Hematoxylin and eosin stain in the pons of *Coq9^+/+^* mice (**g_1_** and **g_2_**), *Coq9^R239X^* mice (**g_3_** and **g_4_**)_,_ and *Coq9^R239X^* mice after PF-4708671 treatment (**g_5_** and **g_6_**). (**h_1_** to **h_6_**) Anti-GFAP stain in the pons of *Coq9^+/+^* mice (**h_1_** and **h_2_**), *Coq9^R239X^* mice (**h_3_** and **h_4_**), and *Coq9^R239X^* mice after PF-4708671 treatment (**h_5_** and **h_6_**). (**i_1_ to i_6_**) Anti-Iba-1 stain in the pons of *Coq9^+/+^* mice (**i_1_** and **i_2_**), *Coq9^R239X^* mice (**i_3_** and **i_4_**), and *Coq9^R239X^* mice after PF-4708671 treatment (**i_5_** and **i_6_**). (**j-m)** Mitochondrial respiration in *Coq9^R239X^* mice after PF-4708671 treatment measured by oxygen consumption rate and the state 3o in brain (**j** and **k**, respectively) and kidney (**l** and **m**, respectively).

Scale bars: 500 μm (**a_1_, b_1,_ a_3_, b_3,_ a_5_, b_5_**); 100 μm (**a_2_, b_2_, a_4_, b_4_, a_6_, b_6_**); 500 μm (**g_1_, h_1_, i_1_, g_3_, h_3_, i_3_, g_5_, h_5_, i_5_**); 100 μm (**g_2_, h_2_, i_2_, g_4_, h_4_, i_4_, g_6_, h_6_, i_6_**).

Data are expressed as mean ± SD. **P* < 0.05; ***P* < 0.01; ****P* < 0.001; *Coq9^+/+^* versus *Coq9^R239X^* or *Coq9^R239X^* after trehalose or PF-4708671 treatment. (one-way ANOVA with a Tukey’s post hoc test or *t*-test).

**SUPPLEMENTAL STATISTIC ANALYSIS**

**Statistic results for Figure 1.**

| **Figure 1a** | | | |
| --- | --- | --- | --- |
| **Tukey's multiple comparisons test** | **Significant?** | **Summary** | ***p* value** |
| 0mo, *Coq9^+/+^* vs. *Coq9^R239X^* | Yes | * | 0.0348 |
| 0mo, *Coq9^+/+^* vs. *Coq9^R239X^* + Rapa28 | Yes | ** | 0.0091 |
| 0mo, *Coq9^+/+^* vs. *Coq9^R239X^* + Rapa225 | Yes | *** | 0.0005 |
| 0mo, *Coq9^R239X^* vs. *Coq9^R239X^* + Rapa28 | No | ns | 0.5979 |
| 0mo, *Coq9^R239X^* vs. *Coq9^R239X^* + Rapa225 | No | ns | 0.3698 |
| 0mo, *Coq9^R239X^* + Rapa28 vs. *Coq9^R239X^* + Rapa225 | No | ns | >0.9999 |
| 1mo, *Coq9^+/+^* vs. *Coq9^R239X^* | Yes | *** | <0.0001 |
| 1mo, *Coq9^+/+^* vs. *Coq9^R239X^* + Rapa28 | Yes | *** | <0.0001 |
| 1mo, *Coq9^+/+^* vs. *Coq9^R239X^* + Rapa225 | Yes | *** | <0.0001 |
| 1mo, *Coq9^R239X^* vs. *Coq9^R239X^* + Rapa28 | No | ns | 0.5161 |
| 1mo, *Coq9^R239X^* vs. *Coq9^R239X^* + Rapa225 | Yes | *** | <0.0001 |
| 1mo, *Coq9^R239X^* + Rapa28 vs. *Coq9^R239X^* + Rapa225 | Yes | *** | <0.0001 |
| 2mo, *Coq9^+/+^* vs. *Coq9^R239X^* | Yes | *** | <0.0001 |
| 2mo, *Coq9^+/+^* vs. *Coq9^R239X^* + Rapa28 | Yes | *** | <0.0001 |
| 2mo, *Coq9^+/+^* vs. *Coq9^R239X^* + Rapa225 | Yes | *** | <0.0001 |
| 2mo, *Coq9^R239X^* vs. *Coq9^R239X^* + Rapa28 | No | ns | 0.6146 |
| 2mo, *Coq9^R239X^* vs. *Coq9^R239X^* + Rapa225 | Yes | *** | <0.0001 |
| 2mo, *Coq9^R239X^* + Rapa28 vs. *Coq9^R239X^* + Rapa225 | Yes | *** | <0.0001 |
| **Figure 1b** | | | |
| **Tukey's multiple comparisons test** | **Significant?** | **Summary** | ***p* value** |
| 0mo, *Coq9^+/+^* vs. *Coq9^R239X^* | Yes | ** | 0.0011 |
| 0mo, *Coq9^+/+^* vs. *Coq9^R239X^* + Rapa28 | No | ns | 0.6416 |
| 0mo, *Coq9^+/+^* vs. *Coq9^R239X^* + Rapa225 | Yes | * | 0.0254 |
| 0mo, *Coq9^R239X^* vs. *Coq9^R239X^* + Rapa28 | No | ns | 0.1336 |
| 0mo, *Coq9^R239X^* vs. *Coq9^R239X^* + Rapa225 | No | ns | 0.6364 |
| 0mo, *Coq9^R239X^* + Rapa28 vs. *Coq9^R239X^* + Rapa225 | No | ns | 0.5872 |
| 1mo, *Coq9^+/+^* vs. *Coq9^R239X^* | Yes | *** | <0.0001 |
| 1mo, *Coq9^+/+^* vs. *Coq9^R239X^* + Rapa28 | Yes | *** | <0.0001 |
| 1mo, *Coq9^+/+^* vs. *Coq9^R239X^* + Rapa225 | Yes | *** | <0.0001 |
| 1mo, *Coq9^R239X^* vs. *Coq9^R239X^* + Rapa28 | No | ns | 0.7200 |
| 1mo, *Coq9^R239X^* vs. *Coq9^R239X^* + Rapa225 | Yes | *** | <0.0001 |
| 1mo, *Coq9^R239X^* + Rapa28 vs. *Coq9^R239X^* + Rapa225 | Yes | *** | <0.0001 |
| 2mo, *Coq9^+/+^* vs. *Coq9^R239X^* | Yes | *** | <0.0001 |
| 2mo, *Coq9^+/+^* vs. *Coq9^R239X^* + Rapa28 | Yes | *** | <0.0001 |
| 2mo, *Coq9^+/+^* vs. *Coq9^R239X^* + Rapa225 | Yes | *** | <0.0001 |
| 2mo, *Coq9^R239X^* vs. *Coq9^R239X^* + Rapa28 | No | ns | 0.5261 |
| 2mo, *Coq9^R239X^* vs. *Coq9^R239X^* + Rapa225 | Yes | *** | <0.0001 |
| 2mo, *Coq9^R239X^* + Rapa28 vs. *Coq9^R239X^* + Rapa225 | Yes | *** | <0.0001 |
| **Figure 1c** | | | |
| **Tukey's multiple comparisons test** | **Significant?** | **Summary** | ***p* value** |
| *Coq9^+/+^* vs. *Coq9^R239X^* | Yes | *** | <0,0001 |
| *Coq9^+/+^* vs. *Coq9^R239X^* + Rapa28 | Yes | *** | 0,0005 |
| *Coq9^+/+^* vs. *Coq9^R239X^* + Rapa225 | Yes | *** | <0,0001 |
| *Coq9^R239X^* vs. *Coq9^R239X^* + Rapa28 | No | ns | 0,1195 |
| *Coq9^R239X^* vs. *Coq9^R239X^* + Rapa225 | No | ns | 0,9992 |
| *Coq9^R239X^* + Rapa28 vs. *Coq9^R239X^* + Rapa225 | No | ns | 0,1380 |
| **Figure 1e** | | | |
| **Log-rank (Mantel-Cox) test** | **Significant?** | **Summary** | ***p* value** |
| *Coq9^+/+^* vs. *Coq9^R239X^* | Yes | *** | <0.0001 |
| *Coq9^+/+^* vs. *Coq9^R239X^* + Rapa28 | Yes | *** | <0.0001 |
| *Coq9^+/+^* vs. *Coq9^R239X^* + Rapa225 | Yes | *** | <0.0001 |
| *Coq9^R239X^* vs. *Coq9^R239X^* + Rapa28 | No | ns | 0.5149 |
| *Coq9^R239X^* vs. *Coq9^R239X^* + Rapa225 | Yes | *** | <0.0001 |
| *Coq9^R239X^* + Rapa28 vs. *Coq9^R239X^* + Rapa225 | Yes | ** | 0.0032 |
| **Gehan-Breslow-Wilcoxon test** | **Significant?** | **Summary** | ***p* value** |
| *Coq9^+/+^* vs. *Coq9^R239X^* | Yes | *** | <0.0001 |
| *Coq9^+/+^* vs. *Coq9^R239X^* + Rapa28 | Yes | *** | <0.0001 |
| *Coq9^+/+^* vs. *Coq9^R239X^* + Rapa225 | Yes | *** | <0.0001 |
| *Coq9^R239X^* vs. *Coq9^R239X^* + Rapa28 | No | ns | 0.3839 |
| *Coq9^R239X^* vs. *Coq9^R239X^* + Rapa225 | Yes | *** | <0.0001 |
| *Coq9^R239X^* + Rapa28 vs. *Coq9^R239X^* + Rapa225 | Yes | ** | 0.0090 |

**Statistic results for Figure 3.**

| **Figure 3a** | | | |
| --- | --- | --- | --- |
| **Tukey's multiple comparisons test** | **Significant?** | **Summary** | ***p* value** |
| *Coq9^+/+^* vs. *Coq9^R239X^* | No | ns | 0.5638 |
| *Coq9^+/+^* vs. *Coq9^R239X^* + Rapa28 | No | ns | >0.9999 |
| *Coq9^+/+^* vs. *Coq9^R239X^* + Rapa225 | No | ns | 0.2285 |
| *Coq9^R239X^* vs. *Coq9^R239X^* + Rapa28 | No | ns | 0.4834 |
| *Coq9^R239X^* vs. *Coq9^R239X^* + Rapa225 | Yes | * | 0.0143 |
| *Coq9^R239X^* + Rapa28 vs. *Coq9^R239X^* + Rapa225 | No | ns | 0.2070 |
| **Figure 3b** | | | |
| **Tukey's multiple comparisons test** | **Significant?** | **Summary** | ***p* value** |
| *Coq9^+/+^* vs. *Coq9^R239X^* | No | ns | 0.9326 |
| *Coq9^+/+^* vs. *Coq9^R239X^* + Rapa28 | No | ns | 0.9994 |
| *Coq9^+/+^* vs. *Coq9^R239X^* + Rapa225 | No | ns | 0.6457 |
| *Coq9^R239X^* vs. *Coq9^R239X^* + Rapa28 | No | ns | 0.9640 |
| *Coq9^R239X^* vs. *Coq9^R239X^* + Rapa225 | No | ns | 0.2778 |
| *Coq9^R239X^* + Rapa28 vs. *Coq9^R239X^* + Rapa225 | No | ns | 0.5745 |
| **Figure 3c** | | | |
| **Tukey's multiple comparisons test** | **Significant?** | **Summary** | ***p* value** |
| *Coq9^+/+^* vs. *Coq9^R239X^* | No | ns | 0.1047 |
| *Coq9^+/+^* vs. *Coq9^R239X^* + Rapa28 | No | ns | 0.9861 |
| *Coq9^+/+^* vs. *Coq9^R239X^* + Rapa225 | Yes | ** | 0.0014 |
| *Coq9^R239X^* vs. *Coq9^R239X^* + Rapa28 | No | ns | 0.4121 |
| *Coq9^R239X^* vs. *Coq9^R239X^* + Rapa225 | Yes | *** | <0.0001 |
| *Coq9^R239X^* + Rapa28 vs. *Coq9^R239X^* + Rapa225 | Yes | ** | 0.0032 |
| **Figure 3d** | | | |
| **Tukey's multiple comparisons test** | **Significant?** | **Summary** | ***p* value** |
| *Coq9^+/+^* vs. *Coq9^R239X^* | No | ns | >0.9999 |
| *Coq9^+/+^* vs. *Coq9^R239X^* + Rapa28 | No | ns | 0.9543 |
| *Coq9^+/+^* vs. *Coq9^R239X^* + Rapa225 | Yes | * | 0.0168 |
| *Coq9^R239X^* vs. *Coq9^R239X^* + Rapa28 | No | ns | 0.9337 |
| *Coq9^R239X^* vs. *Coq9^R239X^* + Rapa225 | Yes | * | 0.0118 |
| *Coq9^R239X^* + Rapa28 vs. *Coq9^R239X^* + Rapa225 | No | ns | 0.0633 |
| **Figure 3e** | | | |
| **Tukey's multiple comparisons test** | **Significant?** | **Summary** | ***p* value** |
| p62, *Coq9^+/+^* vs. *Coq9^R239X^* | No | ns | 0.4038 |
| p62, *Coq9^+/+^* vs. *Coq9^R239X^* + Rapa28 | No | ns | 0.9638 |
| p62, *Coq9^+/+^* vs. *Coq9^R239X^* + Rapa225 | No | ns | 0.9969 |
| p62, *Coq9^R239X^* vs. *Coq9^R239X^* + Rapa28 | No | ns | 0.2670 |
| p62, *Coq9^R239X^* vs. *Coq9^R239X^* + Rapa225 | No | ns | 0.6551 |
| p62, *Coq9^R239X^* + Rapa28 vs. *Coq9^R239X^* + Rapa225 | No | ns | 0.9272 |
| LC3II/LC3I, *Coq9^+/+^* vs. *Coq9^R239X^* | No | ns | 0.7472 |
| LC3II/LC3I, *Coq9^+/+^* vs. *Coq9^R239X^* + Rapa28 | No | ns | 0.1771 |
| LC3II/LC3I, *Coq9^+/+^* vs. *Coq9^R239X^* + Rapa225 | No | ns | 0.7799 |
| LC3II/LC3I, *Coq9^R239X^* vs. *Coq9^R239X^* + Rapa28 | No | ns | 0.5540 |
| LC3II/LC3I, *Coq9^R239X^* vs. *Coq9^R239X^* + Rapa225 | No | ns | 0.9994 |
| LC3II/LC3I, *Coq9^R239X^* + Rapa28 vs. *Coq9^R239X^* + Rapa225 | No | ns | 0.7210 |
| **Figure 3f** | | | |
| **Tukey's multiple comparisons test** | **Significant?** | **Summary** | ***p* value** |
| p62, *Coq9^+/+^* vs. *Coq9^R239X^* | Yes | * | 0.0142 |
| p62, *Coq9^+/+^* vs. *Coq9^R239X^* + Rapa28 | Yes | * | 0.0422 |
| p62, *Coq9^+/+^* vs. *Coq9^R239X^* + Rapa225 | Yes | * | 0.0165 |
| p62, *Coq9^R239X^* vs. *Coq9^R239X^* + Rapa28 | No | ns | 0.9998 |
| p62, *Coq9^R239X^* vs. *Coq9^R239X^* + Rapa225 | No | ns | 0.9553 |
| p62, *Coq9^R239X^* + Rapa28 vs. *Coq9^R239X^* + Rapa225 | No | ns | 0.9815 |
| LC3II/LC3I, *Coq9^+/+^* vs. *Coq9^R239X^* | No | ns | >0.9999 |
| LC3II/LC3I, *Coq9^+/+^* vs. *Coq9^R239X^* + Rapa28 | No | ns | 0.3192 |
| LC3II/LC3I, *Coq9^+/+^* vs. *Coq9^R239X^* + Rapa225 | No | ns | 0.1212 |
| LC3II/LC3I, *Coq9^R239X^* vs. *Coq9^R239X^* + Rapa28 | No | ns | 0.2904 |
| LC3II/LC3I, *Coq9^R239X^* vs. *Coq9^R239X^* + Rapa225 | No | ns | 0.0991 |
| LC3II/LC3I, *Coq9^R239X^* + Rapa28 vs. *Coq9^R239X^* + Rapa225 | Yes | ** | 0.0061 |
| **Figure 3g** | | | |
| **Tukey's multiple comparisons test** | **Significant?** | **Summary** | ***p* value** |
| p62, *Coq9^+/+^* vs. *Coq9^R239X^* | No | ns | 0.2469 |
| p62, *Coq9^+/+^* vs. *Coq9^R239X^* + Rapa28 | No | ns | 0.7277 |
| p62, *Coq9^+/+^* vs. *Coq9^R239X^* + Rapa225 | No | ns | 0.9973 |
| p62, *Coq9^R239X^* vs. *Coq9^R239X^* + Rapa28 | No | ns | 0.0515 |
| p62, *Coq9^R239X^* vs. *Coq9^R239X^* + Rapa225 | No | ns | 0.2695 |
| p62, *Coq9^R239X^* + Rapa28 vs. *Coq9^R239X^* + Rapa225 | No | ns | 0.8703 |
| LC3II/LC3I, *Coq9^+/+^* vs. *Coq9^R239X^* | No | ns | 0.4102 |
| LC3II/LC3I, *Coq9^+/+^* vs. *Coq9^R239X^* + Rapa28 | Yes | * | 0.0348 |
| LC3II/LC3I, *Coq9^+/+^* vs. *Coq9^R239X^* + Rapa225 | No | ns | 0.0877 |
| LC3II/LC3I, *Coq9^R239X^* vs. *Coq9^R239X^* + Rapa28 | No | ns | 0.3560 |
| LC3II/LC3I, *Coq9^R239X^* vs. *Coq9^R239X^* + Rapa225 | No | ns | 0.6186 |
| LC3II/LC3I, *Coq9^R239X^* + Rapa28 vs. *Coq9^R239X^* + Rapa225 | No | ns | 0.9786 |
| **Figure 3h** | | | |
| **Tukey's multiple comparisons test** | **Significant?** | **Summary** | ***p* value** |
| p62, *Coq9^+/+^* vs. *Coq9^R239X^* | No | ns | 0.0521 |
| p62, *Coq9^+/+^* vs. *Coq9^R239X^* + Rapa28 | No | ns | 0.1752 |
| p62, *Coq9^+/+^* vs. *Coq9^R239X^* + Rapa225 | No | ns | 0.8496 |
| p62, *Coq9^R239X^* vs. *Coq9^R239X^* + Rapa28 | No | ns | 0.9984 |
| p62, *Coq9^R239X^* vs. *Coq9^R239X^* + Rapa225 | No | ns | 0.4359 |
| p62, *Coq9^R239X^* + Rapa28 vs. *Coq9^R239X^* + Rapa225 | No | ns | 0.6434 |
| LC3II/LC3I, *Coq9^+/+^* vs. *Coq9^R239X^* | No | ns | 0.8876 |
| LC3II/LC3I, *Coq9^+/+^* vs. *Coq9^R239X^* + Rapa28 | No | ns | 0.1246 |
| LC3II/LC3I, *Coq9^+/+^* vs. *Coq9^R239X^* + Rapa225 | No | ns | 0.9224 |
| LC3II/LC3I, *Coq9^R239X^* vs. *Coq9^R239X^* + Rapa28 | No | ns | 0.3089 |
| LC3II/LC3I, *Coq9^R239X^* vs. *Coq9^R239X^* + Rapa225 | No | ns | >0.9999 |
| LC3II/LC3I, *Coq9^R239X^* + Rapa28 vs. *Coq9^R239X^* + Rapa225 | No | ns | 0.4451 |

**Statistic results for Figure 5.**

| **Figure 5a** | | | |
| --- | --- | --- | --- |
| **Tukey's multiple comparisons test** | **Significant?** | **Summary** | ***p* value** |
| FAD, *Coq9^+/+^* vs. *Coq9^R239X^* | No | ns | 0,3904 |
| FAD, *Coq9^+/+^* vs. *Coq9^R239X^* + Rapa28 | Yes | * | 0,0191 |
| FAD, *Coq9^R239X^* vs. *Coq9^R239X^* + Rapa28 | No | ns | 0,1601 |
| cAMP, *Coq9^+/+^* vs. *Coq9^R239X^* | No | ns | 0.3390 |
| cAMP, *Coq9^+/+^* vs. *Coq9^R239X^* + Rapa28 | No | ns | 0.4558 |
| cAMP, *Coq9^R239X^* vs. *Coq9^R239X^* + Rapa28 | No | ns | 0.9682 |
| UDP-N-acetyl-D-galactosamine, *Coq9^+/+^* vs. *Coq9^R239X^* | No | ns | 0.0667 |
| UDP-N-acetyl-D-galactosamine, *Coq9^+/+^* vs. *Coq9^R239X^* + Rapa28 | No | ns | 0.0543 |
| UDP-N-acetyl-D-galactosamine, *Coq9^R239X^* vs. *Coq9^R239X^* + Rapa28 | No | ns | 0.9905 |
| 5-Hydroxyindoleacetic acid, *Coq9^+/+^* vs. *Coq9^R239X^* | Yes | *** | 0.0004 |
| 5-Hydroxyindoleacetic acid, *Coq9^+/+^* vs. *Coq9^R239X^* + Rapa28 | Yes | * | 0.0119 |
| 5-Hydroxyindoleacetic acid, *Coq9^R239X^* vs. *Coq9^R239X^* + Rapa28 | No | ns | 0.0824 |
| Corticosterone, *Coq9^+/+^* vs. *Coq9^R239X^* | Yes | * | 0.0179 |
| Corticosterone, *Coq9^+/+^* vs. *Coq9^R239X^* + Rapa28 | Yes | * | 0.0265 |
| Corticosterone, *Coq9^R239X^* vs. *Coq9^R239X^* + Rapa28 | No | ns | 0.9650 |
| Cortexolone, *Coq9^+/+^* vs. *Coq9^R239X^* | Yes | *** | <0.0001 |
| Cortexolone, *Coq9^+/+^* vs. *Coq9^R239X^* + Rapa28 | Yes | *** | <0.0001 |
| Cortexolone, *Coq9^R239X^* vs. *Coq9^R239X^* + Rapa28 | No | ns | 0.8223 |
| D-Galactose, *Coq9^+/+^* vs. *Coq9^R239X^* | Yes | * | 0.0146 |
| D-Galactose, *Coq9^+/+^* vs. *Coq9^R239X^* + Rapa28 | No | ns | 0.0979 |
| D-Galactose, *Coq9^R239X^* vs. *Coq9^R239X^* + Rapa28 | No | ns | 0.4661 |
| Gentisic acid, *Coq9^+/+^* vs. *Coq9^R239X^* | No | ns | 0.9709 |
| Gentisic acid, *Coq9^+/+^* vs. *Coq9^R239X^* + Rapa28 | No | ns | 0.2865 |
| Gentisic acid, *Coq9^R239X^* vs. *Coq9^R239X^* + Rapa28 | No | ns | 0.3317 |
| Glutathione, oxidized, *Coq9^+/+^* vs. *Coq9^R239X^* | Yes | ** | 0.0040 |
| Glutathione, oxidized, *Coq9^+/+^* vs. *Coq9^R239X^* + Rapa28 | Yes | * | 0.0141 |
| Glutathione, oxidized, *Coq9^R239X^* vs. *Coq9^R239X^* + Rapa28 | No | ns | 0.6808 |
| Glutathione, *Coq9^+/+^* vs. *Coq9^R239X^* | No | ns | 0.2601 |
| Glutathione, *Coq9^+/+^* vs. *Coq9^R239X^* + Rapa28 | No | ns | 0.4179 |
| Glutathione, *Coq9^R239X^* vs. *Coq9^R239X^* + Rapa28 | No | ns | 0.9139 |
| Hydroxyphenyllactic acid, *Coq9^+/+^* vs. *Coq9^R239X^* | Yes | *** | <0.0001 |
| Hydroxyphenyllactic acid, *Coq9^+/+^* vs. *Coq9^R239X^* + Rapa28 | Yes | *** | 0.0001 |
| Hydroxyphenyllactic acid, *Coq9^R239X^* vs. *Coq9^R239X^* + Rapa28 | No | ns | 0.5712 |
| Riboflavin (vitamin B2), *Coq9^+/+^* vs. *Coq9^R239X^* | No | ns | 0.3233 |
| Riboflavin (vitamin B2), *Coq9^+/+^* vs. *Coq9^R239X^* + Rapa28 | No | ns | 0.0877 |
| Riboflavin (vitamin B2), *Coq9^R239X^* vs. *Coq9^R239X^* + Rapa28 | No | ns | 0.6531 |
| Niacinamide, *Coq9^+/+^* vs. *Coq9^R239X^* | No | ns | 0.8435 |
| Niacinamide, *Coq9^+/+^* vs. *Coq9^R239X^* + Rapa28 | No | ns | 0.8435 |
| Niacinamide, *Coq9^R239X^* vs. *Coq9^R239X^* + Rapa28 | No | ns | >0.9999 |
| L-Ascorbic acid, *Coq9^+/+^* vs. *Coq9^R239X^* | No | ns | 0.7908 |
| L-Ascorbic acid, *Coq9^+/+^* vs. *Coq9^R239X^* + Rapa28 | No | ns | 0.8912 |
| L-Ascorbic acid, *Coq9^R239X^* vs. *Coq9^R239X^* + Rapa28 | No | ns | 0.9780 |
| Pantothenic acid, *Coq9^+/+^* vs. *Coq9^R239X^* | Yes | ** | 0.0017 |
| Pantothenic acid, *Coq9^+/+^* vs. *Coq9^R239X^* + Rapa28 | Yes | ** | 0.0063 |
| Pantothenic acid, *Coq9^R239X^* vs. *Coq9^R239X^* + Rapa28 | No | ns | 0.6187 |
| Citric acid, *Coq9^+/+^* vs. *Coq9^R239X^* | No | ns | 0.7973 |
| Citric acid, *Coq9^+/+^* vs. *Coq9^R239X^* + Rapa28 | No | ns | 0.3264 |
| Citric acid, *Coq9^R239X^* vs. *Coq9^R239X^* + Rapa28 | No | ns | 0.6701 |
| Fumaric acid, *Coq9^+/+^* vs. *Coq9^R239X^* | No | ns | 0.8209 |
| Fumaric acid, *Coq9^+/+^* vs. *Coq9^R239X^* + Rapa28 | No | ns | 0.9367 |
| Fumaric acid, *Coq9^R239X^* vs. *Coq9^R239X^* + Rapa28 | No | ns | 0.9637 |
| Leucine enkephalin, *Coq9^+/+^* vs. *Coq9^R239X^* | No | ns | 0.1500 |
| Leucine enkephalin, *Coq9^+/+^* vs. *Coq9^R239X^* + Rapa28 | No | ns | 0.6838 |
| Leucine enkephalin, *Coq9^R239X^* vs. *Coq9^R239X^* + Rapa28 | No | ns | 0.4678 |
| 8-Amino Caprylic acid, *Coq9^+/+^* vs. *Coq9^R239X^* | No | ns | 0.6427 |
| 8-Amino Caprylic acid, *Coq9^+/+^* vs. *Coq9^R239X^* + Rapa28 | No | ns | 0.1519 |
| 8-Amino Caprylic acid, *Coq9^R239X^* vs. *Coq9^R239X^* + Rapa28 | No | ns | 0.5105 |
| Gamma-Glu-Leu, *Coq9^+/+^* vs. *Coq9^R239X^* | Yes | ** | 0.0057 |
| Gamma-Glu-Leu, *Coq9^+/+^* vs. *Coq9^R239X^* + Rapa28 | Yes | ** | 0.0012 |
| Gamma-Glu-Leu, *Coq9^R239X^* vs. *Coq9^R239X^* + Rapa28 | No | ns | 0.5398 |
| Creatine, *Coq9^+/+^* vs. *Coq9^R239X^* | No | ns | 0.3386 |
| Creatine, *Coq9^+/+^* vs. *Coq9^R239X^* + Rapa28 | No | ns | 0.9987 |
| Creatine, *Coq9^R239X^* vs. *Coq9^R239X^* + Rapa28 | No | ns | 0.3181 |
| Pyroglutamic acid, *Coq9^+/+^* vs. *Coq9^R239X^* | Yes | *** | 0.0007 |
| Pyroglutamic acid, *Coq9^+/+^* vs. *Coq9^R239X^* + Rapa28 | Yes | ** | 0.0043 |
| Pyroglutamic acid, *Coq9^R239X^* vs. *Coq9^R239X^* + Rapa28 | No | ns | 0.3913 |
| Spermidine, *Coq9^+/+^* vs. *Coq9^R239X^* | Yes | ** | 0.0086 |
| Spermidine, *Coq9^+/+^* vs. *Coq9^R239X^* + Rapa28 | Yes | * | 0.0424 |
| Spermidine, *Coq9^R239X^* vs. *Coq9^R239X^* + Rapa28 | No | ns | 0.5718 |
| L-Valine, *Coq9^+/+^* vs. *Coq9^R239X^* | Yes | *** | <0.0001 |
| L-Valine, *Coq9^+/+^* vs. *Coq9^R239X^* + Rapa28 | Yes | *** | <0.0001 |
| L-Valine, *Coq9^R239X^* vs. *Coq9^R239X^* + Rapa28 | No | ns | 0.9985 |
| 2-Amino-3-methyl-1-butanol, *Coq9^+/+^* vs. *Coq9^R239X^* | Yes | *** | 0.0003 |
| 2-Amino-3-methyl-1-butanol, *Coq9^+/+^* vs. *Coq9^R239X^* + Rapa28 | Yes | *** | 0.0002 |
| 2-Amino-3-methyl-1-butanol, *Coq9^R239X^* vs. *Coq9^R239X^* + Rapa28 | No | ns | 0.6934 |
| Indoleacetaldehyde, *Coq9^+/+^* vs. *Coq9^R239X^* | Yes | * | 0.0363 |
| Indoleacetaldehyde, *Coq9^+/+^* vs. *Coq9^R239X^* + Rapa28 | No | ns | 0.2864 |
| Indoleacetaldehyde, *Coq9^R239X^* vs. *Coq9^R239X^* + Rapa28 | No | ns | 0.3921 |
| 3-Hydroxyanthranilic acid, *Coq9^+/+^* vs. *Coq9^R239X^* | Yes | *** | <0.0001 |
| 3-Hydroxyanthranilic acid, *Coq9^+/+^* vs. *Coq9^R239X^* + Rapa28 | Yes | *** | <0.0001 |
| 3-Hydroxyanthranilic acid, *Coq9^R239X^* vs. *Coq9^R239X^* + Rapa28 | No | ns | >0.9999 |
| N-Acetyl-DL-tryptophan, *Coq9^+/+^* vs. *Coq9^R239X^* | No | ns | 0.0852 |
| N-Acetyl-DL-tryptophan, *Coq9^+/+^* vs. *Coq9^R239X^* + Rapa28 | No | ns | 0.1006 |
| N-Acetyl-DL-tryptophan, *Coq9^R239X^* vs. *Coq9^R239X^* + Rapa28 | No | ns | 0.9936 |
| L-tryptophan, *Coq9^+/+^* vs. *Coq9^R239X^* | Yes | * | 0.0149 |
| L-tryptophan, *Coq9^+/+^* vs. *Coq9^R239X^* + Rapa28 | No | ns | 0.2090 |
| L-tryptophan, *Coq9^R239X^* vs. *Coq9^R239X^* + Rapa28 | No | ns | 0.2477 |
| Phenylacetylglycine, *Coq9^+/+^* vs. *Coq9^R239X^* | Yes | *** | 0.0009 |
| Phenylacetylglycine, *Coq9^+/+^* vs. *Coq9^R239X^* + Rapa28 | Yes | *** | 0.0006 |
| Phenylacetylglycine, *Coq9^R239X^* vs. *Coq9^R239X^* + Rapa28 | No | ns | 0.8353 |
| Taurine, *Coq9^+/+^* vs. *Coq9^R239X^* | No | ns | 0.0615 |
| Taurine, *Coq9^+/+^* vs. *Coq9^R239X^* + Rapa28 | No | ns | 0.7090 |
| Taurine, *Coq9^R239X^* vs. *Coq9^R239X^* + Rapa28 | No | ns | 0.2075 |
| L-Aspartic Acid, *Coq9^+/+^* vs. *Coq9^R239X^* | No | ns | 0.5134 |
| L-Aspartic Acid, *Coq9^+/+^* vs. *Coq9^R239X^* + Rapa28 | No | ns | 0.2395 |
| L-Aspartic Acid, *Coq9^R239X^* vs. *Coq9^R239X^* + Rapa28 | No | ns | 0.8192 |
| N-Methylglutamic acid, *Coq9^+/+^* vs. *Coq9^R239X^* | Yes | * | 0.0396 |
| N-Methylglutamic acid, *Coq9^+/+^* vs. *Coq9^R239X^* + Rapa28 | Yes | ** | 0.0031 |
| N-Methylglutamic acid, *Coq9^R239X^* vs. *Coq9^R239X^* + Rapa28 | No | ns | 0.1934 |
| N-Acetyl-L-glutamic acid, *Coq9^+/+^* vs. *Coq9^R239X^* | Yes | ** | 0.0054 |
| N-Acetyl-L-glutamic acid, *Coq9^+/+^* vs. *Coq9^R239X^* + Rapa28 | Yes | ** | 0.0066 |
| N-Acetyl-L-glutamic acid, *Coq9^R239X^* vs. *Coq9^R239X^* + Rapa28 | No | ns | 0.9906 |
| L-Glutamate , *Coq9^+/+^* vs. *Coq9^R239X^* | Yes | * | 0.0493 |
| L-Glutamate , *Coq9^+/+^* vs. *Coq9^R239X^* + Rapa28 | No | ns | 0.1135 |
| L-Glutamate , *Coq9^R239X^* vs. *Coq9^R239X^* + Rapa28 | No | ns | 0.8540 |
| Uric acid, *Coq9^+/+^* vs. *Coq9^R239X^* | No | ns | 0.8976 |
| Uric acid, *Coq9^+/+^* vs. *Coq9^R239X^* + Rapa28 | No | ns | 0.9862 |
| Uric acid, *Coq9^R239X^* vs. *Coq9^R239X^* + Rapa28 | No | ns | 0.8625 |
| Uracil, *Coq9^+/+^* vs. *Coq9^R239X^* | Yes | ** | 0.0066 |
| Uracil, *Coq9^+/+^* vs. *Coq9^R239X^* + Rapa28 | Yes | * | 0.0492 |
| Uracil, *Coq9^R239X^* vs. *Coq9^R239X^* + Rapa28 | No | ns | 0.4016 |
| Thymidine, *Coq9^+/+^* vs. *Coq9^R239X^* | Yes | * | 0.0335 |
| Thymidine, *Coq9^+/+^* vs. *Coq9^R239X^* + Rapa28 | No | ns | 0.0669 |
| Thymidine, *Coq9^R239X^* vs. *Coq9^R239X^* + Rapa28 | No | ns | 0.8982 |
| 5-Deoxy-5-(methylthio)adenosine, *Coq9^+/+^* vs. *Coq9^R239X^* | No | ns | 0.9971 |
| 5-Deoxy-5-(methylthio)adenosine, *Coq9^+/+^* vs. *Coq9^R239X^* + Rapa28 | No | ns | 0.8850 |
| 5-Deoxy-5-(methylthio)adenosine, *Coq9^R239X^* vs. *Coq9^R239X^* + Rapa28 | No | ns | 0.8505 |
| Adenosine, *Coq9^+/+^* vs. *Coq9^R239X^* | No | ns | 0.2800 |
| Adenosine, *Coq9^+/+^* vs. *Coq9^R239X^* + Rapa28 | No | ns | 0.3591 |
| Adenosine, *Coq9^R239X^* vs. *Coq9^R239X^* + Rapa28 | No | ns | 0.9802 |
| Adenine, *Coq9^+/+^* vs. *Coq9^R239X^* | No | ns | 0.0651 |
| Adenine, *Coq9^+/+^* vs. *Coq9^R239X^* + Rapa28 | No | ns | 0.1343 |
| Adenine, *Coq9^R239X^* vs. *Coq9^R239X^* + Rapa28 | No | ns | 0.8857 |
| Guanidylic acid (guanosine monophosphate), *Coq9^+/+^* vs. *Coq9^R239X^* | No | ns | 0.7124 |
| Guanidylic acid (guanosine monophosphate), *Coq9^+/+^* vs. *Coq9^R239X^* + Rapa28 | No | ns | 0.8613 |
| Guanidylic acid (guanosine monophosphate), *Coq9^R239X^* vs. *Coq9^R239X^* + Rapa28 | No | ns | 0.4175 |
| Guanosine, *Coq9^+/+^* vs. *Coq9^R239X^* | No | ns | 0.1706 |
| Guanosine, *Coq9^+/+^* vs. *Coq9^R239X^* + Rapa28 | No | ns | 0.2697 |
| Guanosine, *Coq9^R239X^* vs. *Coq9^R239X^* + Rapa28 | No | ns | 0.9444 |
| Inosine, *Coq9^+/+^* vs. *Coq9^R239X^* | No | ns | 0.8653 |
| Inosine, *Coq9^+/+^* vs. *Coq9^R239X^* + Rapa28 | No | ns | 0.9922 |
| Inosine, *Coq9^R239X^* vs. *Coq9^R239X^* + Rapa28 | No | ns | 0.9044 |
| Hypoxanthine, *Coq9^+/+^* vs. *Coq9^R239X^* | No | ns | 0.3891 |
| Hypoxanthine, *Coq9^+/+^* vs. *Coq9^R239X^* + Rapa28 | No | ns | 0.1679 |
| Hypoxanthine, *Coq9^R239X^* vs. *Coq9^R239X^* + Rapa28 | No | ns | 0.8724 |
| Xanthine, *Coq9^+/+^* vs. *Coq9^R239X^* | No | ns | 0.3071 |
| Xanthine, *Coq9^+/+^* vs. *Coq9^R239X^* + Rapa28 | No | ns | 0.4750 |
| Xanthine, *Coq9^R239X^* vs. *Coq9^R239X^* + Rapa28 | No | ns | 0.9335 |
| Uridine monophosphate (UMP), *Coq9^+/+^* vs. *Coq9^R239X^* | No | ns | 0.1910 |
| Uridine monophosphate (UMP), *Coq9^+/+^* vs. *Coq9^R239X^* + Rapa28 | No | ns | 0.6097 |
| Uridine monophosphate (UMP), *Coq9^R239X^* vs. *Coq9^R239X^* + Rapa28 | No | ns | 0.6325 |
| 5-Methylcytidine, *Coq9^+/+^* vs. *Coq9^R239X^* | No | ns | 0.6410 |
| 5-Methylcytidine, *Coq9^+/+^* vs. *Coq9^R239X^* + Rapa28 | No | ns | 0.9166 |
| 5-Methylcytidine, *Coq9^R239X^* vs. *Coq9^R239X^* + Rapa28 | No | ns | 0.8410 |
| Cytidine, *Coq9^+/+^* vs. *Coq9^R239X^* | No | ns | 0.1224 |
| Cytidine, *Coq9^+/+^* vs. *Coq9^R239X^* + Rapa28 | No | ns | 0.1290 |
| Cytidine, *Coq9^R239X^* vs. *Coq9^R239X^* + Rapa28 | No | ns | 0.9993 |
| Methyl jasmonate, *Coq9^+/+^* vs. *Coq9^R239X^* | No | ns | 0.6785 |
| Methyl jasmonate, *Coq9^+/+^* vs. *Coq9^R239X^* + Rapa28 | No | ns | 0.0909 |
| Methyl jasmonate, *Coq9^R239X^* vs. *Coq9^R239X^* + Rapa28 | No | ns | 0.3152 |
| Farnesal, *Coq9^+/+^* vs. *Coq9^R239X^* | No | ns | >0.9999 |
| Farnesal, *Coq9^+/+^* vs. *Coq9^R239X^* + Rapa28 | No | ns | 0.8941 |
| Farnesal, *Coq9^R239X^* vs. *Coq9^R239X^* + Rapa28 | No | ns | 0.8882 |
| (R)-2-Hydroxycaprylic acid, *Coq9^+/+^* vs. *Coq9^R239X^* | Yes | *** | 0.0001 |
| (R)-2-Hydroxycaprylic acid, *Coq9^+/+^* vs. *Coq9^R239X^* + Rapa28 | Yes | ** | 0.0042 |
| (R)-2-Hydroxycaprylic acid, *Coq9^R239X^* vs. *Coq9^R239X^* + Rapa28 | Yes | * | 0.0490 |
| Taurocholic acid, *Coq9^+/+^* vs. *Coq9^R239X^* | No | ns | 0.2394 |
| Taurocholic acid, *Coq9^+/+^* vs. *Coq9^R239X^* + Rapa28 | No | ns | 0.2595 |
| Taurocholic acid, *Coq9^R239X^* vs. *Coq9^R239X^* + Rapa28 | No | ns | 0.9981 |
| Lignoceric acid, *Coq9^+/+^* vs. *Coq9^R239X^* | No | ns | 0.9990 |
| Lignoceric acid, *Coq9^+/+^* vs. *Coq9^R239X^* + Rapa28 | No | ns | 0.9959 |
| Lignoceric acid, *Coq9^R239X^* vs. *Coq9^R239X^* + Rapa28 | No | ns | 0.9990 |
| Elaidic Acid, *Coq9^+/+^* vs. *Coq9^R239X^* | No | ns | 0.5659 |
| Elaidic Acid, *Coq9^+/+^* vs. *Coq9^R239X^* + Rapa28 | No | ns | 0.7657 |
| Elaidic Acid, *Coq9^R239X^* vs. *Coq9^R239X^* + Rapa28 | No | ns | 0.9374 |
| Linoleic acid, *Coq9^+/+^* vs. *Coq9^R239X^* | No | ns | 0.9307 |
| Linoleic acid, *Coq9^+/+^* vs. *Coq9^R239X^* + Rapa28 | No | ns | 0.8099 |
| Linoleic acid, *Coq9^R239X^* vs. *Coq9^R239X^* + Rapa28 | No | ns | 0.9626 |
| Docosahexaenoic acid, *Coq9^+/+^* vs. *Coq9^R239X^* | No | ns | 0.8982 |
| Docosahexaenoic acid, *Coq9^+/+^* vs. *Coq9^R239X^* + Rapa28 | No | ns | 0.8982 |
| Docosahexaenoic acid, *Coq9^R239X^* vs. *Coq9^R239X^* + Rapa28 | No | ns | 0.6204 |
| Arachidonic Acid, *Coq9^+/+^* vs. *Coq9^R239X^* | No | ns | 0.8907 |
| Arachidonic Acid, *Coq9^+/+^* vs. *Coq9^R239X^* + Rapa28 | No | ns | 0.7739 |
| Arachidonic Acid, *Coq9^R239X^* vs. *Coq9^R239X^* + Rapa28 | No | ns | 0.9712 |
| cis-9-palmitoleic acid, *Coq9^+/+^* vs. *Coq9^R239X^* | Yes | * | 0.0185 |
| cis-9-palmitoleic acid, *Coq9^+/+^* vs. *Coq9^R239X^* + Rapa28 | No | ns | 0.1778 |
| cis-9-palmitoleic acid, *Coq9^R239X^* vs. *Coq9^R239X^* + Rapa28 | No | ns | 0.3487 |
| Eicosapentaenoic Acid, *Coq9^+/+^* vs. *Coq9^R239X^* | Yes | ** | 0.0011 |
| Eicosapentaenoic Acid, *Coq9^+/+^* vs. *Coq9^R239X^* + Rapa28 | Yes | ** | 0.0029 |
| Eicosapentaenoic Acid, *Coq9^R239X^* vs. *Coq9^R239X^* + Rapa28 | No | ns | 0.6537 |
| Myristic acid, *Coq9^+/+^* vs. *Coq9^R239X^* | No | ns | 0.0667 |
| Myristic acid, *Coq9^+/+^* vs. *Coq9^R239X^* + Rapa28 | No | ns | 0.1792 |
| Myristic acid, *Coq9^R239X^* vs. *Coq9^R239X^* + Rapa28 | No | ns | 0.7961 |
| **Figure 5b** | | | |
| **Tukey's multiple comparisons test** | **Significant?** | **Summary** | ***p* value** |
| FAD, *Coq9^+/+^* vs. *Coq9^R239X^* | Yes | * | 0.0138 |
| FAD, *Coq9^+/+^* vs. *Coq9^R239X^* + Rapa28 | Yes | ** | 0.0048 |
| FAD, *Coq9^R239X^* vs. *Coq9^R239X^* + Rapa28 | No | ns | 0.7647 |
| cAMP, *Coq9^+/+^* vs. *Coq9^R239X^* | Yes | ** | 0.0039 |
| cAMP, *Coq9^+/+^* vs. *Coq9^R239X^* + Rapa28 | Yes | *** | 0.0007 |
| cAMP, *Coq9^R239X^* vs. *Coq9^R239X^* + Rapa28 | No | ns | 0.4107 |
| UDP-N-acetyl-D-galactosamine, *Coq9^+/+^* vs. *Coq9^R239X^* | No | ns | 0.1411 |
| UDP-N-acetyl-D-galactosamine, *Coq9^+/+^* vs. *Coq9^R239X^* + Rapa28 | Yes | * | 0.0138 |
| UDP-N-acetyl-D-galactosamine, *Coq9^R239X^* vs. *Coq9^R239X^* + Rapa28 | No | ns | 0.3330 |
| 5-Hydroxyindoleacetic acid, *Coq9^+/+^* vs. *Coq9^R239X^* | No | ns | 0.0702 |
| 5-Hydroxyindoleacetic acid, *Coq9^+/+^* vs. *Coq9^R239X^* + Rapa28 | No | ns | 0.1930 |
| 5-Hydroxyindoleacetic acid, *Coq9^R239X^* vs. *Coq9^R239X^* + Rapa28 | No | ns | 0.6543 |
| Corticosterone, *Coq9^+/+^* vs. *Coq9^R239X^* | Yes | ** | 0.0077 |
| Corticosterone, *Coq9^+/+^* vs. *Coq9^R239X^* + Rapa28 | Yes | ** | 0.0091 |
| Corticosterone, *Coq9^R239X^* vs. *Coq9^R239X^* + Rapa28 | No | ns | 0.9925 |
| Cortisol, *Coq9^+/+^* vs. *Coq9^R239X^* | Yes | ** | 0.0011 |
| Cortisol, *Coq9^+/+^* vs. *Coq9^R239X^* + Rapa28 | Yes | *** | 0.0010 |
| Cortisol, *Coq9^R239X^* vs. *Coq9^R239X^* + Rapa28 | No | ns | 0.9954 |
| UDP-glucose, *Coq9^+/+^* vs. *Coq9^R239X^* | No | ns | 0.0654 |
| UDP-glucose, *Coq9^+/+^* vs. *Coq9^R239X^* + Rapa28 | Yes | ** | 0.0094 |
| UDP-glucose, *Coq9^R239X^* vs. *Coq9^R239X^* + Rapa28 | No | ns | 0.4522 |
| Lactobionic acid, *Coq9^+/+^* vs. *Coq9^R239X^* | Yes | * | 0.0292 |
| Lactobionic acid, *Coq9^+/+^* vs. *Coq9^R239X^* + Rapa28 | Yes | ** | 0.0048 |
| Lactobionic acid, *Coq9^R239X^* vs. *Coq9^R239X^* + Rapa28 | No | ns | 0.2385 |
| Galactonic acid, *Coq9^+/+^* vs. *Coq9^R239X^* | No | ns | 0.0542 |
| Galactonic acid, *Coq9^+/+^* vs. *Coq9^R239X^* + Rapa28 | Yes | * | 0.0477 |
| Galactonic acid, *Coq9^R239X^* vs. *Coq9^R239X^* + Rapa28 | No | ns | 0.9964 |
| D-Galactose, *Coq9^+/+^* vs. *Coq9^R239X^* | No | ns | 0.2155 |
| D-Galactose, *Coq9^+/+^* vs. *Coq9^R239X^* + Rapa28 | No | ns | 0.0586 |
| D-Galactose, *Coq9^R239X^* vs. *Coq9^R239X^* + Rapa28 | No | ns | 0.6752 |
| Glutathione, oxidized, *Coq9^+/+^* vs. *Coq9^R239X^* | No | ns | 0.1357 |
| Glutathione, oxidized, *Coq9^+/+^* vs. *Coq9^R239X^* + Rapa28 | Yes | * | 0.0464 |
| Glutathione, oxidized, *Coq9^R239X^* vs. *Coq9^R239X^* + Rapa28 | No | ns | 0.7716 |
| Glutathione, *Coq9^+/+^* vs. *Coq9^R239X^* | No | ns | 0.4533 |
| Glutathione, *Coq9^+/+^* vs. *Coq9^R239X^* + Rapa28 | No | ns | 0.2160 |
| Glutathione, *Coq9^R239X^* vs. *Coq9^R239X^* + Rapa28 | No | ns | 0.8388 |
| Hydroxyphenyllactic acid, *Coq9^+/+^* vs. *Coq9^R239X^* | No | ns | 0.3078 |
| Hydroxyphenyllactic acid, *Coq9^+/+^* vs. *Coq9^R239X^* + Rapa28 | No | ns | 0.0616 |
| Hydroxyphenyllactic acid, *Coq9^R239X^* vs. *Coq9^R239X^* + Rapa28 | No | ns | 0.3632 |
| Riboflavin (vitamin B2), *Coq9^+/+^* vs. *Coq9^R239X^* | Yes | ** | 0.0036 |
| Riboflavin (vitamin B2), *Coq9^+/+^* vs. *Coq9^R239X^* + Rapa28 | Yes | * | 0.0240 |
| Riboflavin (vitamin B2), *Coq9^R239X^* vs. *Coq9^R239X^* + Rapa28 | No | ns | 0.4426 |
| Niacinamide, *Coq9^+/+^* vs. *Coq9^R239X^* | No | ns | 0.9941 |
| Niacinamide, *Coq9^+/+^* vs. *Coq9^R239X^* + Rapa28 | No | ns | 0.3128 |
| Niacinamide, *Coq9^R239X^* vs. *Coq9^R239X^* + Rapa28 | No | ns | 0.2724 |
| Threonate, *Coq9^+/+^* vs. *Coq9^R239X^* | Yes | *** | 0.0003 |
| Threonate, *Coq9^+/+^* vs. *Coq9^R239X^* + Rapa28 | Yes | *** | 0.0003 |
| Threonate, *Coq9^R239X^* vs. *Coq9^R239X^* + Rapa28 | No | ns | 0.9939 |
| L-Ascorbic acid, *Coq9^+/+^* vs. *Coq9^R239X^* | No | ns | 0.9252 |
| L-Ascorbic acid, *Coq9^+/+^* vs. *Coq9^R239X^* + Rapa28 | No | ns | 0.5341 |
| L-Ascorbic acid, *Coq9^R239X^* vs. *Coq9^R239X^* + Rapa28 | No | ns | 0.3430 |
| Pantothenic acid, *Coq9^+/+^* vs. *Coq9^R239X^* | No | ns | 0.7572 |
| Pantothenic acid, *Coq9^+/+^* vs. *Coq9^R239X^* + Rapa28 | No | ns | 0.7572 |
| Pantothenic acid, *Coq9^R239X^* vs. *Coq9^R239X^* + Rapa28 | No | ns | 0.3608 |
| Citric acid, *Coq9^+/+^* vs. *Coq9^R239X^* | No | ns | 0.4050 |
| Citric acid, *Coq9^+/+^* vs. *Coq9^R239X^* + Rapa28 | No | ns | 0.2316 |
| Citric acid, *Coq9^R239X^* vs. *Coq9^R239X^* + Rapa28 | No | ns | 0.8865 |
| Fumaric acid, *Coq9^+/+^* vs. *Coq9^R239X^* | No | ns | 0.5805 |
| Fumaric acid, *Coq9^+/+^* vs. *Coq9^R239X^* + Rapa28 | No | ns | 0.1662 |
| Fumaric acid, *Coq9^R239X^* vs. *Coq9^R239X^* + Rapa28 | No | ns | 0.6059 |
| Succinic acid semialdehyde, *Coq9^+/+^* vs. *Coq9^R239X^* | Yes | * | 0.0184 |
| Succinic acid semialdehyde, *Coq9^+/+^* vs. *Coq9^R239X^* + Rapa28 | Yes | ** | 0.0096 |
| Succinic acid semialdehyde, *Coq9^R239X^* vs. *Coq9^R239X^* + Rapa28 | No | ns | 0.8712 |
| 4-acetamidobutanoate, *Coq9^+/+^* vs. *Coq9^R239X^* | No | ns | 0.8900 |
| 4-acetamidobutanoate, *Coq9^+/+^* vs. *Coq9^R239X^* + Rapa28 | No | ns | 0.1814 |
| 4-acetamidobutanoate, *Coq9^R239X^* vs. *Coq9^R239X^* + Rapa28 | No | ns | 0.3428 |
| N-Acetyl-L-Histidine, *Coq9^+/+^* vs. *Coq9^R239X^* | No | ns | 0.1705 |
| N-Acetyl-L-Histidine, *Coq9^+/+^* vs. *Coq9^R239X^* + Rapa28 | No | ns | 0.1240 |
| N-Acetyl-L-Histidine, *Coq9^R239X^* vs. *Coq9^R239X^* + Rapa28 | No | ns | 0.9751 |
| S-Adenosylhomocysteine, *Coq9^+/+^* vs. *Coq9^R239X^* | No | ns | 0.8791 |
| S-Adenosylhomocysteine, *Coq9^+/+^* vs. *Coq9^R239X^* + Rapa28 | No | ns | 0.6476 |
| S-Adenosylhomocysteine, *Coq9^R239X^* vs. *Coq9^R239X^* + Rapa28 | No | ns | 0.3821 |
| 8-Amino Caprylic acid, *Coq9^+/+^* vs. *Coq9^R239X^* | No | ns | 0.5789 |
| 8-Amino Caprylic acid, *Coq9^+/+^* vs. *Coq9^R239X^* + Rapa28 | No | ns | 0.9875 |
| 8-Amino Caprylic acid, *Coq9^R239X^* vs. *Coq9^R239X^* + Rapa28 | No | ns | 0.4936 |
| Gamma-Glu-Leu, *Coq9^+/+^* vs. *Coq9^R239X^* | No | ns | 0.5310 |
| Gamma-Glu-Leu, *Coq9^+/+^* vs. *Coq9^R239X^* + Rapa28 | No | ns | 0.3887 |
| Gamma-Glu-Leu, *Coq9^R239X^* vs. *Coq9^R239X^* + Rapa28 | No | ns | 0.0793 |
| Pyroglutamic acid , *Coq9^+/+^* vs. *Coq9^R239X^* | Yes | * | 0.0380 |
| Pyroglutamic acid , *Coq9^+/+^* vs. *Coq9^R239X^* + Rapa28 | No | ns | 0.1767 |
| Pyroglutamic acid , *Coq9^R239X^* vs. *Coq9^R239X^* + Rapa28 | No | ns | 0.5937 |
| Spermidine, *Coq9^+/+^* vs. *Coq9^R239X^* | Yes | * | 0.0486 |
| Spermidine, *Coq9^+/+^* vs. *Coq9^R239X^* + Rapa28 | Yes | * | 0.0355 |
| Spermidine, *Coq9^R239X^* vs. *Coq9^R239X^* + Rapa28 | No | ns | 0.9779 |
| L-Norleucine, *Coq9^+/+^* vs. *Coq9^R239X^* | No | ns | 0.4393 |
| L-Norleucine, *Coq9^+/+^* vs. *Coq9^R239X^* + Rapa28 | No | ns | 0.2812 |
| L-Norleucine, *Coq9^R239X^* vs. *Coq9^R239X^* + Rapa28 | No | ns | 0.9342 |
| L-Valine, *Coq9^+/+^* vs. *Coq9^R239X^* | Yes | *** | 0.0007 |
| L-Valine, *Coq9^+/+^* vs. *Coq9^R239X^* + Rapa28 | Yes | *** | 0.0003 |
| L-Valine, *Coq9^R239X^* vs. *Coq9^R239X^* + Rapa28 | No | ns | 0.6567 |
| 2-Amino-3-methyl-1-butanol, *Coq9^+/+^* vs. *Coq9^R239X^* | No | ns | 0.3888 |
| 2-Amino-3-methyl-1-butanol, *Coq9^+/+^* vs. *Coq9^R239X^* + Rapa28 | No | ns | 0.3075 |
| 2-Amino-3-methyl-1-butanol, *Coq9^R239X^* vs. *Coq9^R239X^* + Rapa28 | Yes | * | 0.0390 |
| Indoleacetaldehyde, *Coq9^+/+^* vs. *Coq9^R239X^* | No | ns | 0.0587 |
| Indoleacetaldehyde, *Coq9^+/+^* vs. *Coq9^R239X^* + Rapa28 | Yes | * | 0.0179 |
| Indoleacetaldehyde, *Coq9^R239X^* vs. *Coq9^R239X^* + Rapa28 | No | ns | 0.6641 |
| 5-Methoxyindoleacetate, *Coq9^+/+^* vs. *Coq9^R239X^* | No | ns | 0.6349 |
| 5-Methoxyindoleacetate, *Coq9^+/+^* vs. *Coq9^R239X^* + Rapa28 | No | ns | 0.9184 |
| 5-Methoxyindoleacetate, *Coq9^R239X^* vs. *Coq9^R239X^* + Rapa28 | No | ns | 0.4164 |
| 3-Hydroxyanthranilic acid, *Coq9^+/+^* vs. *Coq9^R239X^* | Yes | *** | 0.0006 |
| 3-Hydroxyanthranilic acid, *Coq9^+/+^* vs. *Coq9^R239X^* + Rapa28 | Yes | *** | 0.0007 |
| 3-Hydroxyanthranilic acid, *Coq9^R239X^* vs. *Coq9^R239X^* + Rapa28 | No | ns | 0.9984 |
| N-Acetyl-L-phenylalanine, *Coq9^+/+^* vs. *Coq9^R239X^* | No | ns | 0.0809 |
| N-Acetyl-L-phenylalanine, *Coq9^+/+^* vs. *Coq9^R239X^* + Rapa28 | Yes | * | 0.0208 |
| N-Acetyl-L-phenylalanine, *Coq9^R239X^* vs. *Coq9^R239X^* + Rapa28 | No | ns | 0.6701 |
| Phenylacetylglycine, *Coq9^+/+^* vs. *Coq9^R239X^* | No | ns | 0.3133 |
| Phenylacetylglycine, *Coq9^+/+^* vs. *Coq9^R239X^* + Rapa28 | No | ns | 0.3402 |
| Phenylacetylglycine, *Coq9^R239X^* vs. *Coq9^R239X^* + Rapa28 | No | ns | 0.9978 |
| Taurine, *Coq9^+/+^* vs. *Coq9^R239X^* | No | ns | 0.0813 |
| Taurine, *Coq9^+/+^* vs. *Coq9^R239X^* + Rapa28 | No | ns | 0.0755 |
| Taurine, *Coq9^R239X^* vs. *Coq9^R239X^* + Rapa28 | No | ns | 0.9987 |
| L-Aspartic Acid, *Coq9^+/+^* vs. *Coq9^R239X^* | No | ns | 0.0544 |
| L-Aspartic Acid, *Coq9^+/+^* vs. *Coq9^R239X^* + Rapa28 | No | ns | 0.1332 |
| L-Aspartic Acid, *Coq9^R239X^* vs. *Coq9^R239X^* + Rapa28 | No | ns | 0.8326 |
| N-Methylglutamic acid, *Coq9^+/+^* vs. *Coq9^R239X^* | Yes | *** | 0.0007 |
| N-Methylglutamic acid, *Coq9^+/+^* vs. *Coq9^R239X^* + Rapa28 | Yes | *** | 0.0002 |
| N-Methylglutamic acid, *Coq9^R239X^* vs. *Coq9^R239X^* + Rapa28 | No | ns | 0.6804 |
| N-Acetyl-L-glutamic acid, *Coq9^+/+^* vs. *Coq9^R239X^* | No | ns | 0.5660 |
| N-Acetyl-L-glutamic acid, *Coq9^+/+^* vs. *Coq9^R239X^* + Rapa28 | No | ns | 0.9841 |
| N-Acetyl-L-glutamic acid, *Coq9^R239X^* vs. *Coq9^R239X^* + Rapa28 | No | ns | 0.6658 |
| L-Glutamate , *Coq9^+/+^* vs. *Coq9^R239X^* | No | ns | 0.1740 |
| L-Glutamate , *Coq9^+/+^* vs. *Coq9^R239X^* + Rapa28 | No | ns | 0.1467 |
| L-Glutamate , *Coq9^R239X^* vs. *Coq9^R239X^* + Rapa28 | No | ns | 0.9926 |
| Uric acid, *Coq9^+/+^* vs. *Coq9^R239X^* | Yes | * | 0.0135 |
| Uric acid, *Coq9^+/+^* vs. *Coq9^R239X^* + Rapa28 | Yes | ** | 0.0044 |
| Uric acid, *Coq9^R239X^* vs. *Coq9^R239X^* + Rapa28 | No | ns | 0.8737 |
| Thymidine, *Coq9^+/+^* vs. *Coq9^R239X^* | No | ns | 0.0903 |
| Thymidine, *Coq9^+/+^* vs. *Coq9^R239X^* + Rapa28 | No | ns | 0.0775 |
| Thymidine, *Coq9^R239X^* vs. *Coq9^R239X^* + Rapa28 | No | ns | 0.9899 |
| 5-Deoxy-5-(methylthio)adenosine, *Coq9^+/+^* vs. *Coq9^R239X^* | No | ns | 0.0603 |
| 5-Deoxy-5-(methylthio)adenosine, *Coq9^+/+^* vs. *Coq9^R239X^* + Rapa28 | Yes | * | 0.0271 |
| 5-Deoxy-5-(methylthio)adenosine, *Coq9^R239X^* vs. *Coq9^R239X^* + Rapa28 | No | ns | 0.8666 |
| Adenosine, *Coq9^+/+^* vs. *Coq9^R239X^* | No | ns | 0.1327 |
| Adenosine, *Coq9^+/+^* vs. *Coq9^R239X^* + Rapa28 | Yes | ** | 0.0079 |
| Adenosine, *Coq9^R239X^* vs. *Coq9^R239X^* + Rapa28 | No | ns | 0.2113 |
| Adenine, *Coq9^+/+^* vs. *Coq9^R239X^* | No | ns | 0.5014 |
| Adenine, *Coq9^+/+^* vs. *Coq9^R239X^* + Rapa28 | Yes | * | 0.0255 |
| Adenine, *Coq9^R239X^* vs. *Coq9^R239X^* + Rapa28 | No | ns | 0.1538 |
| deoxyguanosine 5’-monophosphate (dGMP), *Coq9^+/+^* vs. *Coq9^R239X^* | Yes | * | 0.0228 |
| deoxyguanosine 5’-monophosphate (dGMP), *Coq9^+/+^* vs. *Coq9^R239X^* + Rapa28 | Yes | ** | 0.0026 |
| deoxyguanosine 5’-monophosphate (dGMP), *Coq9^R239X^* vs. *Coq9^R239X^* + Rapa28 | No | ns | 0.3431 |
| Guanosine, *Coq9^+/+^* vs. *Coq9^R239X^* | No | ns | 0.9763 |
| Guanosine, *Coq9^+/+^* vs. *Coq9^R239X^* + Rapa28 | No | ns | 0.9498 |
| Guanosine, *Coq9^R239X^* vs. *Coq9^R239X^* + Rapa28 | No | ns | 0.8082 |
| Guanine, *Coq9^+/+^* vs. *Coq9^R239X^* | No | ns | 0.2884 |
| Guanine, *Coq9^+/+^* vs. *Coq9^R239X^* + Rapa28 | Yes | ** | 0.0025 |
| Guanine, *Coq9^R239X^* vs. *Coq9^R239X^* + Rapa28 | Yes | * | 0.0152 |
| Inosine, *Coq9^+/+^* vs. *Coq9^R239X^* | No | ns | 0.2122 |
| Inosine, *Coq9^+/+^* vs. *Coq9^R239X^* + Rapa28 | Yes | * | 0.0111 |
| Inosine, *Coq9^R239X^* vs. *Coq9^R239X^* + Rapa28 | No | ns | 0.1861 |
| Uridine monophosphate (UMP), *Coq9^+/+^* vs. *Coq9^R239X^* | No | ns | 0.2357 |
| Uridine monophosphate (UMP), *Coq9^+/+^* vs. *Coq9^R239X^* + Rapa28 | Yes | * | 0.0492 |
| Uridine monophosphate (UMP), *Coq9^R239X^* vs. *Coq9^R239X^* + Rapa28 | No | ns | 0.5719 |
| 5-Methylcytidine, *Coq9^+/+^* vs. *Coq9^R239X^* | No | ns | 0.9338 |
| 5-Methylcytidine, *Coq9^+/+^* vs. *Coq9^R239X^* + Rapa28 | No | ns | 0.3278 |
| 5-Methylcytidine, *Coq9^R239X^* vs. *Coq9^R239X^* + Rapa28 | No | ns | 0.2019 |
| Cytidine, *Coq9^+/+^* vs. *Coq9^R239X^* | No | ns | 0.1389 |
| Cytidine, *Coq9^+/+^* vs. *Coq9^R239X^* + Rapa28 | Yes | ** | 0.0064 |
| Cytidine, *Coq9^R239X^* vs. *Coq9^R239X^* + Rapa28 | No | ns | 0.1638 |
| 3-Hydroxy-3-methyl-glutaric acid, *Coq9^+/+^* vs. *Coq9^R239X^* | Yes | * | 0.0353 |
| 3-Hydroxy-3-methyl-glutaric acid, *Coq9^+/+^* vs. *Coq9^R239X^* + Rapa28 | Yes | ** | 0.0018 |
| 3-Hydroxy-3-methyl-glutaric acid, *Coq9^R239X^* vs. *Coq9^R239X^* + Rapa28 | No | ns | 0.0977 |
| 2-Methylhippuric acid, *Coq9^+/+^* vs. *Coq9^R239X^* | No | ns | 0.2433 |
| 2-Methylhippuric acid, *Coq9^+/+^* vs. *Coq9^R239X^* + Rapa28 | No | ns | 0.2299 |
| 2-Methylhippuric acid, *Coq9^R239X^* vs. *Coq9^R239X^* + Rapa28 | No | ns | 0.9991 |
| Methyl linolenate, *Coq9^+/+^* vs. *Coq9^R239X^* | No | ns | 0.1669 |
| Methyl linolenate, *Coq9^+/+^* vs. *Coq9^R239X^* + Rapa28 | Yes | * | 0.0264 |
| Methyl linolenate, *Coq9^R239X^* vs. *Coq9^R239X^* + Rapa28 | No | ns | 0.4837 |
| Farnesal, *Coq9^+/+^* vs. *Coq9^R239X^* | No | ns | 0.4284 |
| Farnesal, *Coq9^+/+^* vs. *Coq9^R239X^* + Rapa28 | No | ns | 0.0758 |
| Farnesal, *Coq9^R239X^* vs. *Coq9^R239X^* + Rapa28 | No | ns | 0.4708 |
| (R)-2-Hydroxycaprylic acid, *Coq9^+/+^* vs. *Coq9^R239X^* | No | ns | 0.1054 |
| (R)-2-Hydroxycaprylic acid, *Coq9^+/+^* vs. *Coq9^R239X^* + Rapa28 | No | ns | 0.1454 |
| (R)-2-Hydroxycaprylic acid, *Coq9^R239X^* vs. *Coq9^R239X^* + Rapa28 | No | ns | 0.9689 |
| Cholic acid, *Coq9^+/+^* vs. *Coq9^R239X^* | No | ns | 0.9907 |
| Cholic acid, *Coq9^+/+^* vs. *Coq9^R239X^* + Rapa28 | No | ns | 0.5120 |
| Cholic acid, *Coq9^R239X^* vs. *Coq9^R239X^* + Rapa28 | No | ns | 0.3928 |
| Taurocholic acid, *Coq9^+/+^* vs. *Coq9^R239X^* | No | ns | 0.8801 |
| Taurocholic acid, *Coq9^+/+^* vs. *Coq9^R239X^* + Rapa28 | No | ns | 0.4275 |
| Taurocholic acid, *Coq9^R239X^* vs. *Coq9^R239X^* + Rapa28 | No | ns | 0.6402 |
| Lignoceric acid, *Coq9^+/+^* vs. *Coq9^R239X^* | Yes | *** | 0.0009 |
| Lignoceric acid, *Coq9^+/+^* vs. *Coq9^R239X^* + Rapa28 | Yes | *** | 0.0003 |
| Lignoceric acid, *Coq9^R239X^* vs. *Coq9^R239X^* + Rapa28 | No | ns | 0.8331 |
| Elaidic Acid, *Coq9^+/+^* vs. *Coq9^R239X^* | No | ns | 0.9656 |
| Elaidic Acid, *Coq9^+/+^* vs. *Coq9^R239X^* + Rapa28 | No | ns | 0.1556 |
| Elaidic Acid, *Coq9^R239X^* vs. *Coq9^R239X^* + Rapa28 | No | ns | 0.1064 |
| Linoleic acid, *Coq9^+/+^* vs. *Coq9^R239X^* | No | ns | 0.3107 |
| Linoleic acid, *Coq9^+/+^* vs. *Coq9^R239X^* + Rapa28 | Yes | ** | 0.0091 |
| Linoleic acid, *Coq9^R239X^* vs. *Coq9^R239X^* + Rapa28 | No | ns | 0.1008 |
| Docosahexaenoic acid, *Coq9^+/+^* vs. *Coq9^R239X^* | Yes | ** | 0.0027 |
| Docosahexaenoic acid, *Coq9^+/+^* vs. *Coq9^R239X^* + Rapa28 | No | ns | 0.1220 |
| Docosahexaenoic acid, *Coq9^R239X^* vs. *Coq9^R239X^* + Rapa28 | Yes | * | 0.0427 |
| Arachidonic Acid, *Coq9^+/+^* vs. *Coq9^R239X^* | No | ns | 0.1030 |
| Arachidonic Acid, *Coq9^+/+^* vs. *Coq9^R239X^* + Rapa28 | Yes | ** | 0.0020 |
| Arachidonic Acid, *Coq9^R239X^* vs. *Coq9^R239X^* + Rapa28 | No | ns | 0.0630 |
| cis-9-palmitoleic acid, *Coq9^+/+^* vs. *Coq9^R239X^* | Yes | * | 0.0440 |
| cis-9-palmitoleic acid, *Coq9^+/+^* vs. *Coq9^R239X^* + Rapa28 | No | ns | 0.8720 |
| cis-9-palmitoleic acid, *Coq9^R239X^* vs. *Coq9^R239X^* + Rapa28 | Yes | * | 0.0202 |
| Eicosapentaenoic Acid, *Coq9^+/+^* vs. *Coq9^R239X^* | Yes | *** | <0.0001 |
| Eicosapentaenoic Acid, *Coq9^+/+^* vs. *Coq9^R239X^* + Rapa28 | Yes | *** | <0.0001 |
| Eicosapentaenoic Acid, *Coq9^R239X^* vs. *Coq9^R239X^* + Rapa28 | Yes | ** | 0.0032 |
| Myristic acid, *Coq9^+/+^* vs. *Coq9^R239X^* | Yes | ** | 0.0026 |
| Myristic acid, *Coq9^+/+^* vs. *Coq9^R239X^* + Rapa28 | No | ns | 0.6287 |
| Myristic acid, *Coq9^R239X^* vs. *Coq9^R239X^* + Rapa28 | Yes | * | 0.0102 |

**Statistical results for Figure 6.**

| **Figure 6a** | | | |
| --- | --- | --- | --- |
| **Tukey's multiple comparisons test** | **Significant?** | **Summary** | ***p* value** |
| *Coq9^+/+^* vs. *Coq9^R239X^* | Yes | *** | 0.0005 |
| *Coq9^+/+^* vs. *Coq9^R239X^* + Rapa28 | Yes | ** | 0.0064 |
| *Coq9^+/+^* vs. *Coq9^R239X^* + Rapa225 | Yes | * | 0.0395 |
| *Coq9^R239X^* vs. *Coq9^R239X^* + Rapa28 | No | ns | 0.9767 |
| *Coq9^R239X^* vs. *Coq9^R239X^* + Rapa225 | No | ns | 0.9865 |
| *Coq9^R239X^* + Rapa28 vs. *Coq9^R239X^* + Rapa225 | No | ns | >0.9999 |
| **Figure 6b** | | | |
| **Tukey's multiple comparisons test** | **Significant?** | **Summary** | ***p* value** |
| *Coq9^+/+^* vs. *Coq9^R239X^* | Yes | *** | <0.0001 |
| *Coq9^+/+^* vs. *Coq9^R239X^* + Rapa28 | Yes | *** | <0.0001 |
| *Coq9^+/+^* vs. *Coq9^R239X^* + Rapa225 | Yes | *** | <0.0001 |
| *Coq9^R239X^* vs. *Coq9^R239X^* + Rapa28 | No | ns | >0.9999 |
| *Coq9^R239X^* vs. *Coq9^R239X^* + Rapa225 | No | ns | >0.9999 |
| *Coq9^R239X^* + Rapa28 vs. *Coq9^R239X^* + Rapa225 | No | ns | >0.9999 |
| **Figure 6c** | | | |
| **Tukey's multiple comparisons test** | **Significant?** | **Summary** | ***p* value** |
| *Coq9^+/+^* vs. *Coq9^R239X^* | Yes | ** | 0.0050 |
| *Coq9^+/+^* vs. *Coq9^R239X^* + Rapa28 | No | ns | 0.0805 |
| *Coq9^+/+^* vs. *Coq9^R239X^* + Rapa225 | No | ns | 0.0742 |
| *Coq9^R239X^* vs. *Coq9^R239X^* + Rapa28 | No | ns | 0.9991 |
| *Coq9^R239X^* vs. *Coq9^R239X^* + Rapa225 | No | ns | 0.9997 |
| *Coq9^R239X^* + Rapa28 vs. *Coq9^R239X^* + Rapa225 | No | ns | >0.9999 |
| **Figure 6d** | | | |
| **Tukey's multiple comparisons test** | **Significant?** | **Summary** | ***p* value** |
| *Coq9^+/+^* vs. *Coq9^R239X^* | Yes | *** | <0.0001 |
| *Coq9^+/+^* vs. *Coq9^R239X^* + Rapa28 | Yes | *** | <0.0001 |
| *Coq9^+/+^* vs. *Coq9^R239X^* + Rapa225 | Yes | *** | <0.0001 |
| *Coq9^R239X^* vs. *Coq9^R239X^* + Rapa28 | No | ns | 0.9995 |
| *Coq9^R239X^* vs. *Coq9^R239X^* + Rapa225 | No | ns | >0.9999 |
| *Coq9^R239X^* + Rapa28 vs. *Coq9^R239X^* + Rapa225 | No | ns | 0.9994 |
| **Figure 6e** | | | |
| **Tukey's multiple comparisons test** | **Significant?** | **Summary** | ***p* value** |
| *Coq9^+/+^* vs. *Coq9^R239X^* | Yes | *** | <0.0001 |
| *Coq9^+/+^* vs. *Coq9^R239X^* + Rapa28 | Yes | *** | <0.0001 |
| *Coq9^+/+^* vs. *Coq9^R239X^* + Rapa225 | Yes | *** | <0.0001 |
| *Coq9^R239X^* vs. *Coq9^R239X^* + Rapa28 | No | ns | >0.9999 |
| *Coq9^R239X^* vs. *Coq9^R239X^* + Rapa225 | No | ns | 0.9964 |
| *Coq9^R239X^* + Rapa28 vs. *Coq9^R239X^* + Rapa225 | No | ns | 0.9950 |
| **Figure 6f** | | | |
| **Tukey's multiple comparisons test** | **Significant?** | **Summary** | ***p* value** |
| *Coq9^+/+^* vs. *Coq9^R239X^* | Yes | *** | 0.0005 |
| *Coq9^+/+^* vs. *Coq9^R239X^* + Rapa28 | Yes | ** | 0.0035 |
| *Coq9^+/+^* vs. *Coq9^R239X^* + Rapa225 | Yes | *** | 0.0001 |
| *Coq9^R239X^* vs. *Coq9^R239X^* + Rapa28 | No | ns | 0.9459 |
| *Coq9^R239X^* vs. *Coq9^R239X^* + Rapa225 | No | ns | 0.1022 |
| *Coq9^R239X^* + Rapa28 vs. *Coq9^R239X^* + Rapa225 | No | ns | 0.0590 |
| **Figure 6g** | | | |
| **Tukey's multiple comparisons test** | **Significant?** | **Summary** | ***p* value** |
| *Coq9^+/+^* vs. *Coq9^R239X^* | Yes | *** | <0.0001 |
| *Coq9^+/+^* vs. *Coq9^R239X^* + Rapa28 | Yes | *** | <0.0001 |
| *Coq9^+/+^* vs. *Coq9^R239X^* + Rapa225 | Yes | *** | <0.0001 |
| *Coq9^R239X^* vs. *Coq9^R239X^* + Rapa28 | No | ns | 0.6663 |
| *Coq9^R239X^* vs. *Coq9^R239X^* + Rapa225 | No | ns | 0.8291 |
| *Coq9^R239X^* + Rapa28 vs. *Coq9^R239X^* + Rapa225 | No | ns | 0.9975 |
| **Figure 6h** | | | |
| **Tukey's multiple comparisons test** | **Significant?** | **Summary** | ***p* value** |
| *Coq9^+/+^* vs. *Coq9^R239X^* | Yes | *** | <0.0001 |
| *Coq9^+/+^* vs. *Coq9^R239X^* + Rapa28 | Yes | *** | <0.0001 |
| *Coq9^+/+^* vs. *Coq9^R239X^* + Rapa225 | Yes | ** | 0.0070 |
| *Coq9^R239X^* vs. *Coq9^R239X^* + Rapa28 | No | ns | 0.5373 |
| *Coq9^R239X^* vs. *Coq9^R239X^* + Rapa225 | No | ns | 0.4268 |
| *Coq9^R239X^* + Rapa28 vs. *Coq9^R239X^* + Rapa225 | No | ns | 0.0839 |
| **Figure 6j** | | | |
| **Tukey's multiple comparisons test** | **Significant?** | **Summary** | ***p* value** |
| *Coq9^+/+^* vs. *Coq9^R239X^* | Yes | *** | <0.0001 |
| *Coq9^+/+^* vs. *Coq9^R239X^* + Rapa28 | Yes | *** | <0.0001 |
| *Coq9^+/+^* vs. *Coq9^R239X^* + Rapa225 | Yes | *** | <0.0001 |
| *Coq9^R239X^* vs. *Coq9^R239X^* + Rapa28 | No | ns | 0.9549 |
| *Coq9^R239X^* vs. *Coq9^R239X^* + Rapa225 | No | ns | 0.9590 |
| *Coq9^R239X^* + Rapa28 vs. *Coq9^R239X^* + Rapa225 | No | ns | 0.8023 |
| **Figure 6k** | | | |
| **Tukey's multiple comparisons test** | **Significant?** | **Summary** | ***p* value** |
| *Coq9^+/+^* vs. *Coq9^R239X^* | Yes | *** | <0.0001 |
| *Coq9^+/+^* vs. *Coq9^R239X^* + Rapa28 | Yes | *** | <0.0001 |
| *Coq9^+/+^* vs. *Coq9^R239X^* + Rapa225 | Yes | *** | <0.0001 |
| *Coq9^R239X^* vs. *Coq9^R239X^* + Rapa28 | No | ns | 0.9996 |
| *Coq9^R239X^* vs. *Coq9^R239X^* + Rapa225 | No | ns | 0.9990 |
| *Coq9^R239X^* + Rapa28 vs. *Coq9^R239X^* + Rapa225 | No | ns | >0.9999 |

**Statistic results for Figure S2.**

| **Figure S2** | | | |
| --- | --- | --- | --- |
| **Tukey's multiple comparisons test** | **Significant?** | **Summary** | ***p* value** |
| *Coq9^+/+^* vs. *Coq9^R239X^* | Yes | * | 0.0115 |
| *Coq9^+/+^* vs. *Coq9^R239X^* + Rapa28 | Yes | * | 0.0213 |
| *Coq9^+/+^* vs. *Coq9^R239X^* + Rapa225 | No | ns | 0.7278 |
| *Coq9^R239X^* vs. *Coq9^R239X^* + Rapa28 | No | ns | 0.9821 |
| *Coq9^R239X^* vs. *Coq9^R239X^* + Rapa225 | No | ns | 0.0532 |
| *Coq9^R239X^* + Rapa28 vs. *Coq9^R239X^* + Rapa225 | No | ns | 0.0987 |

**Statistic results for Figure S3.**

| **Figure S3** | | | |
| --- | --- | --- | --- |
| **t-test** | **Significant?** | **Summary** | ***p* value** |
| p62, *Coq9^+/+^* vs. *Coq9^R239X^* | No | ns | 0.6510 |
| LC3II/LC3I, *Coq9^+/+^* vs. *Coq9^R239X^* | Yes | * | 0.0134 |

**Statistic results for Figure S6.**

| **Figure S6c** | | | |
| --- | --- | --- | --- |
| **Tukey's multiple comparisons test** | **Significant?** | **Summary** | ***p* value** |
| *Coq9^+/+^* vs. *Coq9^R239X^* | Yes | *** | <0.0001 |
| *Coq9^+/+^* vs. *Coq9^R239X^* + Trehalose | Yes | *** | <0.0001 |
| *Coq9^R239X^* vs. *Coq9^R239X^* + Trehalose | No | ns | 0.9919 |
| **Figure S6d** | | | |
| **Tukey's multiple comparisons test** | **Significant?** | **Summary** | ***p* value** |
| *Coq9^+/+^* vs. *Coq9^R239X^* | Yes | ** | 0.0014 |
| *Coq9^+/+^* vs. *Coq9^R239X^* + Trehalose | Yes | ** | 0.0046 |
| *Coq9^R239X^* vs. *Coq9^R239X^* + Trehalose | No | ns | 0.8451 |
| **Figure S6e** | | | |
| **Tukey's multiple comparisons test** | **Significant?** | **Summary** | ***p* value** |
| *Coq9^+/+^* vs. *Coq9^R239X^* | Yes | *** | 0.0001 |
| *Coq9^+/+^* vs. *Coq9^R239X^* + Trehalose | Yes | ** | 0.0028 |
| *Coq9^R239X^* vs. *Coq9^R239X^* + Trehalose | No | ns | 0.1945 |
| **Figure S6f** | | | |
| **Tukey's multiple comparisons test** | **Significant?** | **Summary** | ***p* value** |
| *Coq9^+/+^* vs. *Coq9^R239X^* | No | ns | 0.1823 |
| *Coq9^+/+^* vs. *Coq9^R239X^* + Trehalose | No | ns | 0.0899 |
| *Coq9^R239X^* vs. *Coq9^R239X^* + Trehalose | No | ns | 0.9032 |
| **Figure S6k** | | | |
| **Tukey's multiple comparisons test** | **Significant?** | **Summary** | ***p* value** |
| *Coq9^+/+^* vs. *Coq9^R239X^* | Yes | * | 0.0132 |
| *Coq9^+/+^* vs. *Coq9^R239X^* + PF-4708671 | Yes | * | 0.0107 |
| *Coq9^R239X^* vs. *Coq9^R239X^* + PF-4708671 | No | ns | 0.9795 |
| **Figure S6m** | | | |
| **Tukey's multiple comparisons test** | **Significant?** | **Summary** | ***p* value** |
| *Coq9^+/+^* vs. *Coq9^R239X^* | Yes | ** | 0.0011 |
| *Coq9^+/+^* vs. *Coq9^R239X^* + PF-4708671 | Yes | ** | 0.0015 |
| *Coq9^R239X^* vs. *Coq9^R239X^* + PF-4708671 | No | ns | 0.9765 |
